# Arabidopsis RSS1 Mediates Cross-Talk Between Glucose and Light Signaling During Hypocotyl Elongation Growth

**DOI:** 10.1038/s41598-017-16239-y

**Published:** 2017-11-23

**Authors:** Manjul Singh, Aditi Gupta, Dhriti Singh, Jitendra P. Khurana, Ashverya Laxmi

**Affiliations:** 1 National Institute of Plant Genome Research, New Delhi, 110067 India; 2 Interdisciplinary center for Plant Genomics and Department of Plant Molecular biology, University of Delhi South Campus, New Delhi, 110021 India

## Abstract

Plants possess exuberant plasticity that facilitates its ability to adapt and survive under challenging environmental conditions. The developmental plasticity largely depends upon cellular elongation which is governed by a complex network of environmental and phytohormonal signals. Here, we report role of glucose (Glc) and Glc-regulated factors in controlling elongation growth and shade response in Arabidopsis. Glc controls shade induced hypocotyl elongation in a dose dependent manner. We have identified a Glc repressed factor *REGULATED BY SUGAR AND SHADE1* (*RSS1*) encoding for an atypical basic helix-loop-helix (bHLH) protein of unknown biological function that is required for normal Glc actions. Phenotype analysis of mutant and overexpression lines suggested RSS1 to be a negative regulator of elongation growth. RSS1 affects overall auxin homeostasis. RSS1 interacts with the elongation growth-promoting proteins HOMOLOG OF BEE2 INTERACTING WITH IBH 1 (HBI1) and BR ENHANCED EXPRESSION2 (BEE2) and negatively affects the transcription of their downstream targets such as *YUCs*, *INDOLE-3-ACETIC ACID INDUCIBLE* (*IAAs*), *LONG HYPOCOTYL IN FAR-RED1* (*HFR1*), *HOMEOBOX PROTEIN 2* (*ATHB2*), *XYLOGLUCAN ENDOTRANSGLUCOSYLASE/HYDROLASES* (*XTHs*) and *EXPANSINS*. We propose, Glc signals might maintain optimal hypocotyl elongation under multiple signals such as light, shade and phytohormones through the central growth regulatory bHLH/HLH module.

## Introduction

Plants get challenged in day to day life by various environmental factors and to cope with them they modulate their architecture accordingly. Plants growing in a dense environment compete to acquire maximum resources from their surroundings be it nutrients or light. Under shaded conditions, plants perceive reduction in red (R) to far-red (FR) light ratio (R/FR) that triggers numerous morphological alterations such as hypocotyl elongation, suppression/inhibition of cotyledon expansion, enhanced growth of the stem and upward direction of leaves to escape low light. Plants have different photoreceptors to perceive various spectral regions of light such as phytochromes (PHYs), cryptochromes (CRYs), phototropins, zeitlupes and UVB-RESISTANCE 8 (UVR8)^1^. This diversity in light perception allows plants to respond to a wide range of developmental and physiological processes. Phytochromes sense R and FR light and regulate various developmental processes like seed germination, hypocotyl elongation, cotyledon expansion, plastid development, shade avoidance, flowering and senescence^1,2^. Light perception causes a conformational change in phytochromes and converts them to active Pfr form^3^. These active phytochromes translocate to the nucleus and interact with PHYTOCHROME INTERACTING FACTORS (PIFs) that regulate transcriptional machinery in response to changes in light availability^1,4–6^. CONSTITUTIVE PHOTOMORPHOGENIC1 (COP1), an E3 ubiquitin ligase facilitating targeting and degradation of proteins through 26S proteasome, is also involved in regulating shade avoidance response^7,8^. In shaded conditions, COP1 gets accumulated in the nucleus^9^. A bZIP transcription factor ELONGATED HYPOCOTYL 5 (HY5), which works downstream to most sensory photoreceptors, can also mediate plant adaptation to shade^7^. Upon prolonged exposure to low R/FR, HY5 down regulates early shade induced genes in a PHYA-dependent manner^10^. HY5 has also been shown to inhibit shade induced hypocotyl elongation when plants are exposed to short sunflecks^11^. In contrast, shade has been shown to increase HY5 protein stability and decrease LONG HYPOCOTYL IN FAR-RED1 (HFR1) protein stability suggesting that enhanced nuclear localization of COP1 under shade differentially regulates its targets^8^.

In Arabidopsis, perception of low R/FR leads to expression of several non-DNA binding atypical bHLH factors, which act as negative regulators of shade induced plant growth. These negative regulators which fine tune the elongation growth response towards shade, light and hormones are: HFR1^12^, PHYTOCHROME RAPIDLY REGULATED1 (PAR1) and PAR2^13^, INCREASED LEAF INCLINATION1 binding bHLH 1 (IBH1)^14^ and ILI1 BINDING bHLH1 like-1 (IBL1)^15^. The HFR1 and PAR1 proteins negatively regulate the activity of PIF4 and PIF5 by forming HLH/bHLH heterodimers and inhibiting their binding to the target promoters^12,16^. Apart from direct interactions with PIFs, these regulators also bind to hypocotyl elongation promoting bHLH transcription factors working downstream to PIFs and inhibit their activity. For example, IBH1 interacts with HOMOLOG OF BEE2 INTERACTING WITH IBH1 (HBI1) which directly activates elongation-promoting genes, like *EXPANSINs* and *XYLOGLUCAN ENDOTRANSGLYCOSYLASE/HYDROLASEs* ^17,18^. Regulation of cell elongation through these negative regulators requires a tightly regulated balance between formations of homo- or hetero-dimers. Formations of homo-dimers will titer out the negative regulators whereas formation of hetero-dimer with positive regulators will inactivate the positive regulators^17^. The differential expression levels of different promoting and repressing regulators determine whether a plant should escape or adapt to shade.

Plants fulfil their energy requirement by fixing light into a metabolizable form via photosynthesis where carbohydrates (sugar) are utilized as fuel for growth. For efficient sugar synthesis and energy production, it is required that plants get a desired amount of photosynthetically active radiation (PAR). Under natural conditions, PAR covers a range of 400 nm (blue light) to 700 nm (red light). When under shade, plants perceive more levels of FR light (ca.735 nm) and due to a subsequent reduction in R:FR ratio, a signal is triggered through phytochromes (mainly phyB). Photosynthetically generated sugars, such as sucrose (Suc) and glucose (Glc) not only serve as basic elements regulating cellular metabolism but can also act as signal molecules by a coordinated modulation of gene expression and enzyme activities in both source and sink tissues^19,20^. In recent years, key players in the Glc signaling network have also been identified using Arabidopsis as the model system^19,21–26^. Glc being the second most abundant sugar in Arabidopsis has an important role to play in regulating growth and development^27–29^. Glc, as a signaling molecule, can influence every aspect of plant growth and development ranging from cell proliferation, cell expansion and elongation, seed germination, seedling growth and development and reproduction to senescence^19,20,23,30–32^. Sugars/Glc can act like hormones in translating nutrient status to regulate growth and floral transition^19,28,31,33,34^. Multiple signals, such as light, shade, nutrients and phytohormones integrate and regulate common transcriptional signatures to orchestrate growth and development in plants. Previously, sugar and phytohormone signal cross-talks have been shown to modulate critical growth and developmental processes^19,23–25,35–43^. Here, we have investigated the inter-connections between Glc, light and phytohormone signalling networks during elongation growth under simulated shade. Hypocotyl growth assay is the most common way to explore shade avoidance responses under controlled conditions^6,10,44^. In nature, shade induced signals are perceived in a complex manner where plant distinguishes between the presence of proximal but non-shading neighbours and between direct foliar shade^44^. Under controlled conditions, proximity shade signals can be induced either by supplementing FR light to a source of white light^45^ or by treatment with FR pulse at the end of light cycle (End-of-Day-FR/EOD-FR) to induce SAS^6,46^. Reduced R:FR ratio under shade conditions results in an equilibrium shift of phytochromes toward Pr form triggering SAS^45^. The pulse of EODFR causes the removal of Pfr form at the beginning of dark period resulting in longer hypocotyls relative to non-treated seedlings^5^. In present study, we have used EODFR treatment to study hypocotyl elongation in response to simulated shade. We have also characterized a Glc repressible but shade, Brassinosteroid (BR) and auxin induced gene of unknown function. The candidate gene, based on its sugar and shade mediated transcriptional regulation, was designated as *R* *egulated by* *S* *ugar and* *S* *hade*1 (*RSS1*). RSS1 encodes for atypical bHLH protein that acts as a negative regulator of cell elongation in response to multiple signals such as light, shade, phytohormones and temperature.

## Results

### Effect of Glc on simulated shade mediated hypocotyl elongation growth

**Figure 1:**
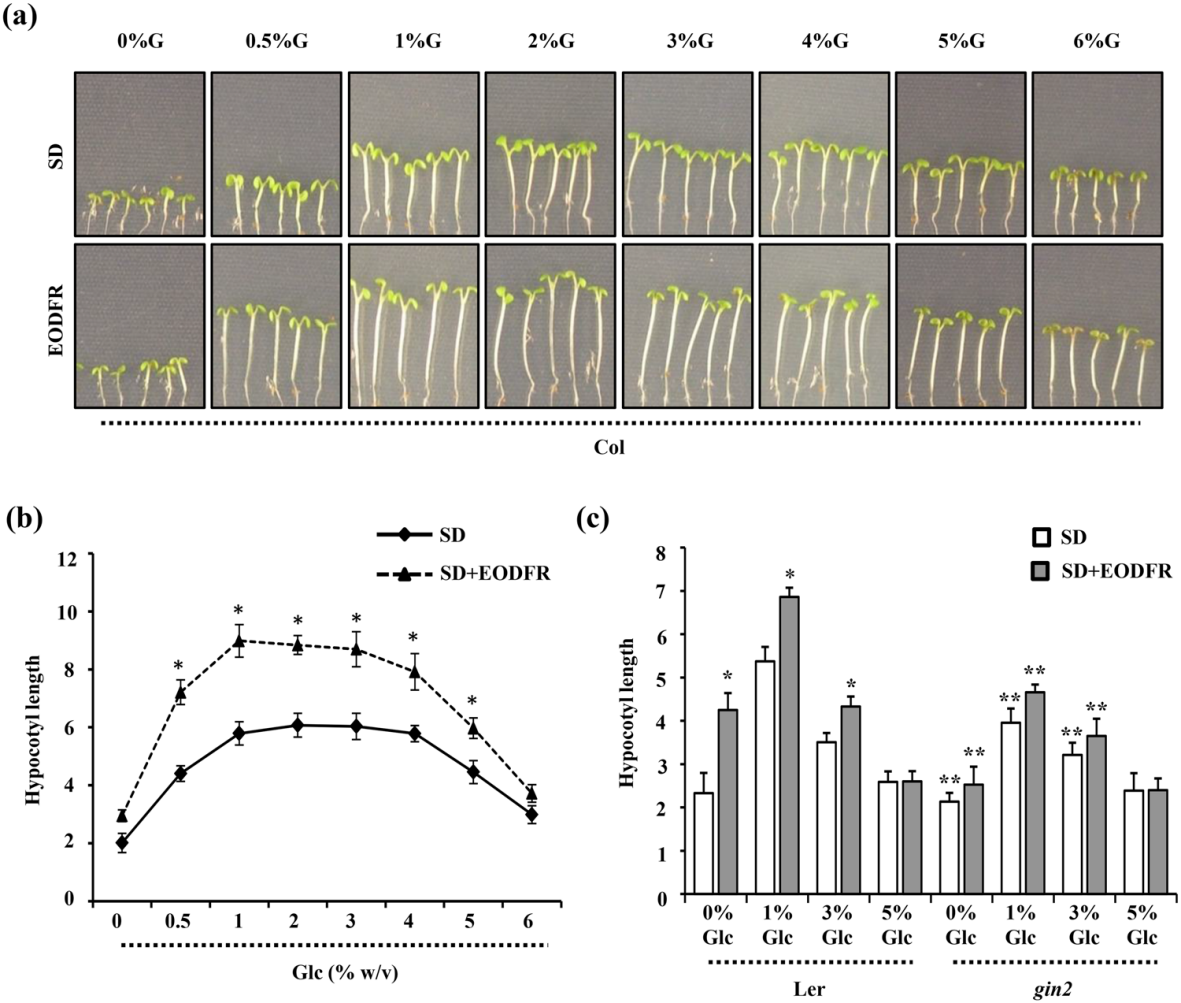
Glc regulates shade induced hypocotyl elongation in Arabidopsis. **(a)** Pictures and **(b)** graph showing effect of increasing Glc concentration on hypocotyl elongation growth under simulated shade conditions. WT (Col-0) seeds were germinated and grown vertically onto ½ MS media supplemented with increasing doses of Glc (0%, 0.5%, 1%, 2%, 3%, 4%, 5% and 6% w/v) for 3d under short day conditions and EODFR treatment was applied from 4^th^ d onwards. Hypocotyl elongation growth of EODFR treated seedlings was compared with that of 6-d-old seedlings growing in control conditions. Lower doses of Glc (0.5% to 2%) promote EODFR induced hypocotyl elongation whereas the response was significantly inhibited in presence of higher Glc concentrations (5%, 6%). **(c)** The HXK1-Glc sensor mutant *gin2*-*1* showed significantly less sensitivity for both Glc and EODFR induced hypocotyl elongation as compared to that of WT, suggesting the involvement of HXK1-dependent Glc signaling in regulating shade-induced hypocotyl elongation growth. Values represent the average of three biological replicates each having 15 seedlings and error bar represents SE. (Student’s t-test; P < 0.01; *control vs. treatment; **WT vs. mutant).

**Figure 2:**
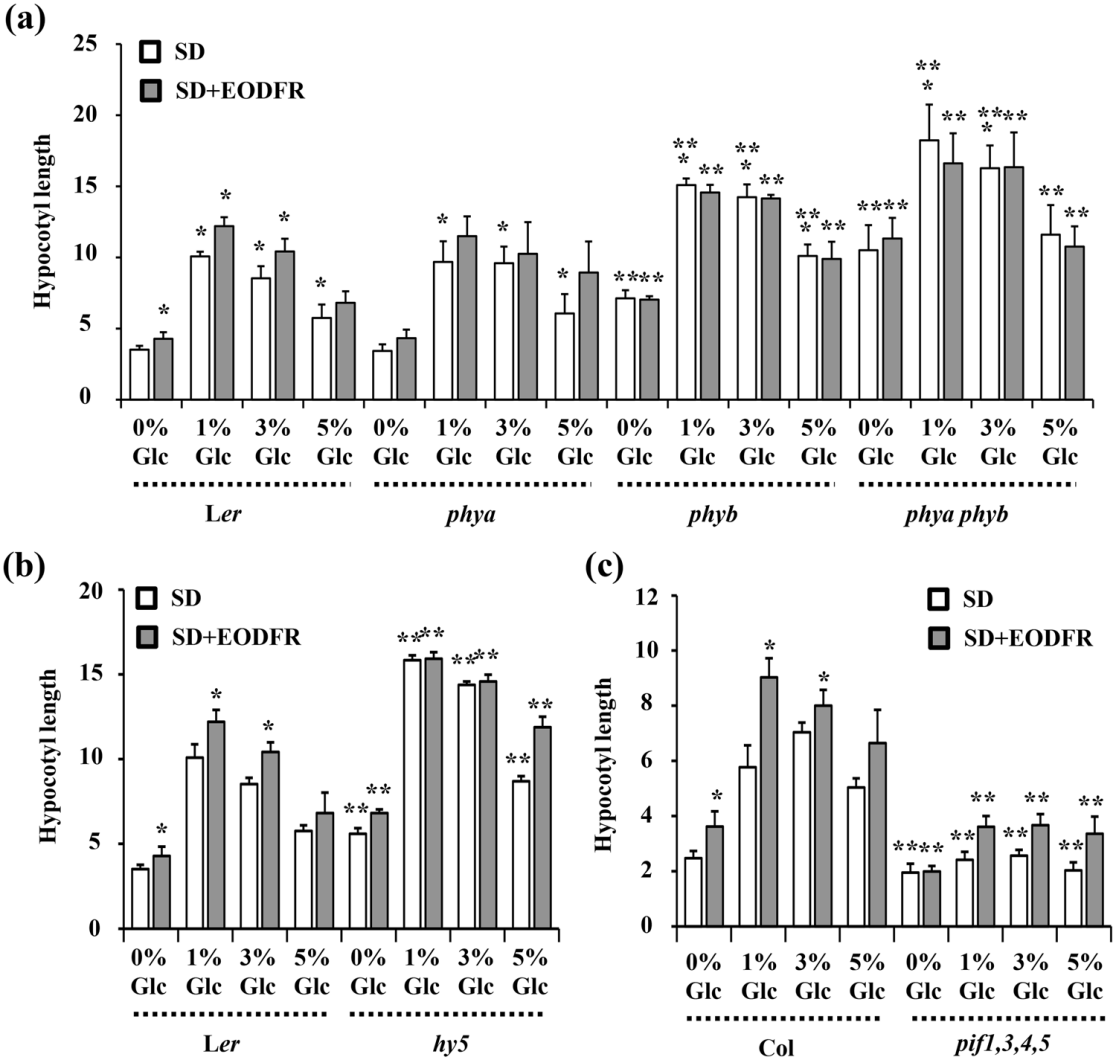
Involvement of various light signaling components in Glc mediated regulation of hypocotyl elongation. 3d old short day grown seedlings of WT (Ler, Col-0) and light signaling mutants (**a**) *phya*, *phyb*, *phya phyb*; (**b**) *hy5*; and (**c**) *pif1345* (*pifq*) were subjected to EODFR treatment in presence or absence of different concentrations of Glc for 3d and hypocotyl elongation growth was measured. The photoreceptor mutants *phya*, *phyb* and *phya phyb* and *hy5* mutant defective in light signaling could respond to Glc-mediated hypocotyl elongation but were found resistant for further EODFR induced hypocotyl elongation at all Glc concentrations. The *pifq* mutant on the other hand showed complete resistance towards both Glc as well as EODFR induced hypocotyl elongation growth. These results suggest that for Glc-modulation of shade response an intact and functional light response pathway is needed. Values represent the average of two biological replicates and error bar represents SE. (Student’s T-test; P < 0.01; *control vs. treatment; **WT vs. mutant).

The hypocotyl elongation growth is under control of a central growth-regulatory circuit that integrates various environmental, developmental and phytohormonal signaling pathways, such as light, shade, temperature, auxin, BR and Gibberellins (GA)^14,47^. Since Glc signaling shows extensive cross-talk with auxin and BR signaling and has a prominent role in regulating hypocotyl elongation growth under both darkness and light^37,39,43^, we hypothesized Glc to be a key regulator of shade induced hypocotyl elongation as well. Previous reports suggested that exogenous sugar could promote plant growth under shaded conditions and HEXOKINASE 1 (HXK1) might also play a role during this response^48,49^. To confirm these hypotheses under our experimental conditions, we checked the EODFR-induced hypocotyl elongation growth of WT (Col-0) seedlings in presence or absence of increasing concentrations of Glc (0%, 0.5%, 1%, 2%, 3%, 4%, 5% and 6% w/v). The lower concentrations of Glc (0.5%, 1% Glc) exerted a promotery effect whereas the higher doses of Glc (6% Glc) did not further enhance EODFR induced hypocotyl elongation (Figs 1a,b, S1a). The HXK1-dependent Glc signaling mutant *glucose insensitive* 2 (*gin2-1*) showed perturbed response with respect to both Glc as well as simulated shade induced hypocotyl elongation growth suggesting HXK1 to be a converging node for shade and Glc signals in Arabidopsis (Figs 1c, S1b,c). The HXK1-independent Glc signaling mutants *regulator of g-protein signaling1* (*rgs1-1*) and *g protein alpha subunit 1* (*gpa1-4*), on the other hand, showed wild type like response towards simulated shade induced hypocotyl elongation growth (Fig. S1b,d,e). Although exogenously applied Glc could regulate simulated shade induced hypocotyl elongation in WT, it could not further enhance the shade mediated hypocotyl elongation in *phytochrome a-201 (phya)*, *phytochrome b-5* (*phyb*), *phya phyb* double mutant and *phytochrome interacting factor1,3,4,5 (pifq)* quadruple mutants, suggesting Glc signaling to be acting downstream of light signaling (Figs 2, S1f). We then compared the global transcript profiles regulated by simulated shade and Glc to define the extent of overlap and nature of interaction between these two signals. Out of 878 simulated shade regulated transcripts (3 h FR/WL; FC ≥2^5^), Glc alone (3% Glc vs 0% Glc w/v; FC ≥2^37^); could regulate 486 (55%) genes transcriptionally. Majority of this overlapping transcriptome (75%) is antagonistically regulated by both these signals (Fig. S2). The inhibitory effect of Glc on shade response was further substantiated by the fact that most of the core shade related genes were down regulated by Glc (Table S1). Further, we checked the Glc mediated transcriptional regulation of the genome targets of AUXIN RESPONSE FACTOR6 (ARF6)^14^, BRASSINAZOLE-RESISTANT 1 (BZR1) and PIF4^50^. Glc could transcriptionally regulate a large proportion of ARF6 binding targets (32%), BZR1 targets (31%) or PIF4 (31%) (Fig. S3). Interestingly, Glc could also transcriptionally regulate 35% of common genomic targets of BZR1-ARF6-PIF4 module and majority of these genes (63%) were repressed by Glc (Fig. S3; Table S2). The hypocotyl cell elongation under shade avoidance response is tightly regulated via cooperative interactions among the tripartite HLH/bHLH module which is formed through antagonistic interactions among DNA-binding bHLH factors, such as PREs with non-DNA-binding HLH factors such as IBH1, HFR1, PAR1 and PAR2^13^. In literature, few atypical bHLH candidates such as HFR1^51^, KIDARI^52^, PACLOBUTRAZOL RESISTANCE1 (PRE1)^53^, PAR1 and PAR2 have been reported to play key regulatory functions in many aspects of growth and development in Arabidopsis. Interestingly, Glc could regulate the transcript levels of many HLH/bHLH factors (Table S3). Collectively, our observations revealed the importance of sugar signaling in modulating elongation growth and shade avoidance responses. We then performed overlap analysis of Glc-shade coregulated genes, auxin responsive genes, common targets of BZR1-ARF6-PIF4 and bHLH factors and identified a single gene (*AT3G29370*) that matched all criteria (Fig. S4). We selected *AT3G29370* as a candidate to study interaction and interdependence of Glc, shade and phytohormone signals in Arabidopsis.

### RSS1 is a Glc and shade regulated atypical bHLH factor

*AT3G29370* encodes a short, 101 amino acid long protein of unknown biological function. Based on literature mining, it was found that *AT3G29370* belonged to novel class of atypical bHLH proteins^54^. Based on the sugar and shade mediated transcriptional regulation, we named *AT3G29370* as *RSS1* (*R* *EGULATED BY* *S* *UGAR AND* *S* *HADE1*). Most of these atypical HLH factors lack essential amino acid residues in the basic region which are required for DNA binding. The functional significance of such specific amino acid replacements in the basic region is still unknown^54^. In RSS1, these essential basic residues have been replaced by Gly. To further confirm this, we aligned RSS1 protein sequence with bHLH and atypical bHLH protein belonging to closest subfamilies using Clustal omega programme (Fig. S5a). It was found that most of the conserved sites were fitting well to the hypothetical, predictive consensus motif previously proposed^52–54^ (Fig. S5b). Based on the alignment pattern, a phylogenetic tree was generated through neighbour-joining method using full-length amino acid sequences via MEGA6 programme with 1000 boot strap values. RSS1 was found to be closely related to HLH3, HLH4, PAR1 and PAR2, while the typical bHLHs such as BEE1, BEE2, BEE3, CRYPTOCHROME-INTERACTING BASIC-HELIX-LOOP-HELIX1 (CIB1), HBI1 etc. were grouped together (Fig. S5b).

**Figure 3:**
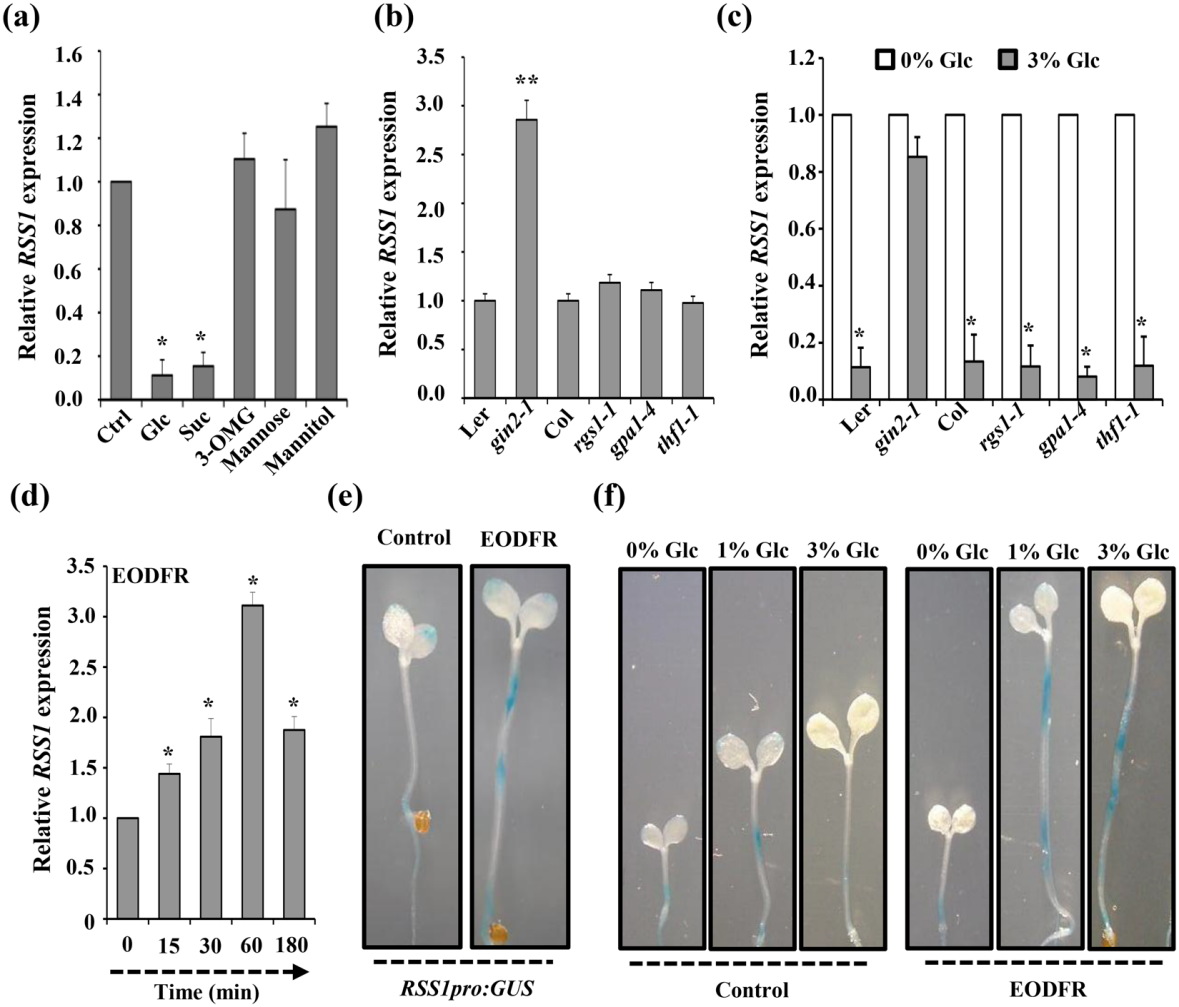
Glc- and simulated shade-mediated transcriptional regulation of *RSS1* in *Arabidopsis*. (**a**) Relative expression of *RSS1* on different sugar treatment shows that *RSS1* is specifically repressed by Glc and sucrose (Suc), seedlings treated with sugar free ½ MS media were considered as control. **(b)** The HXK1-dependent Glc signaling mutant *gin2-1* showed relatively higher *RSS1* transcript accumulation as compared with WT. (**c**) Analysis of Glc mediated repression of *RSS1* transcript levels in HXK1-dependent and HXK1-independent Glc signaling mutants suggested involvement of HXK1-dependent signaling pathway in controlling *RSS1* transcription. (**d**) Effect of EODFR mediated simulated shade conditions on *RSS1* transcript abundance. 3-d-old WT (Col-0) seedlings growing under short day (SD) conditions were exposed to FR light at the end-of the light and RSS1 transcript levels were quantified after various time points. The RSS1 transcripts were significantly induced within 15 min reaching a maximum level at 1 h exposure to EODFR pulse suggesting RSS1 to be an early shade inducible gene. (**e**) The *RSS1pro:GUS* reporter expression was significantly induced upon 1 h EODFR exposure as compared with SD control. (**f**) Increasing Glc concentration could significantly repress *RSS1pro:GUS* reporter expression under control conditions whereas under shaded conditions, *RSS1* expression was enhanced in presence of Glc. Expression values represent the average of two biological replicates with three technical replicates each and error bar represents SE. (Student’s t-test; P < 0.05; *control vs. treatment; **WT vs. mutant).

*RSS1* transcript levels were found to be highly repressed by exogenous Glc treatment (3% w/v; Fig. 3a). To find if other sugars also could exert a similar effect, we checked the effect of various sugar analogs on *RSS1* transcript abundance. Besides Glc, only Suc could repress *RSS1* transcript, whereas no significant change could be observed in response to mannose, mannitol and 3-O-Methylglucose (3-OMG) (Fig. 3a). To find out the involvement of different components of Glc signaling, basal *RSS1* levels as well as Glc-repression of *RSS1* transcript were analysed in mutants of both HXK1-dependent and independent Glc signaling components. The basal *RSS1* transcript level was significantly increased in HXK1-dependent mutant *gin2-1* as compared to the WT (Fig. 3b). Further, treatment with 3% Glc could not repress *RSS1* transcript levels in *gin2-1* (Fig. 3c). Mutants of HXK1-independent components *rgs1-1*, *thylakoid formation1* (*thf1-1*) and *gpa1* did not have any difference in basal *RSS1* levels and no change in Glc repression was observed as compared to WT (Fig. 3b,c). All these results suggest that *RSS1* is regulated through HXK1-dependent Glc signaling pathway.

We also examined the effect of simulated shade condition (EODFR) on *RSS1* transcript abundance. WT (Col-0) seedlings were grown under short-day (SD) conditions and FR light for 1 h was provided at the end of light period. The expression levels of *RSS1* were found to be significantly induced upon EODFR treatment (Fig. 3d). The *RSS1*_*pro*_ *:GUS* expression pattern upon EODFR treatment further substantiated our qRT-PCR results (Fig. 3e). Under short day conditions, exogenous Glc (3% w/v) was able to repress *RSS1*_*pro*_ *:GUS* expression. However, under simulated shade conditions, *RSS1*_*pro*_:*GUS* expression was induced in presence of Glc (Fig. 3f). These results suggest that Glc might differentially regulate *RSS1* expression to regulate hypocotyl elongation growth under different environmental conditions.

### RSS1 is a negative regulator of seedling development

**Figure 4:**
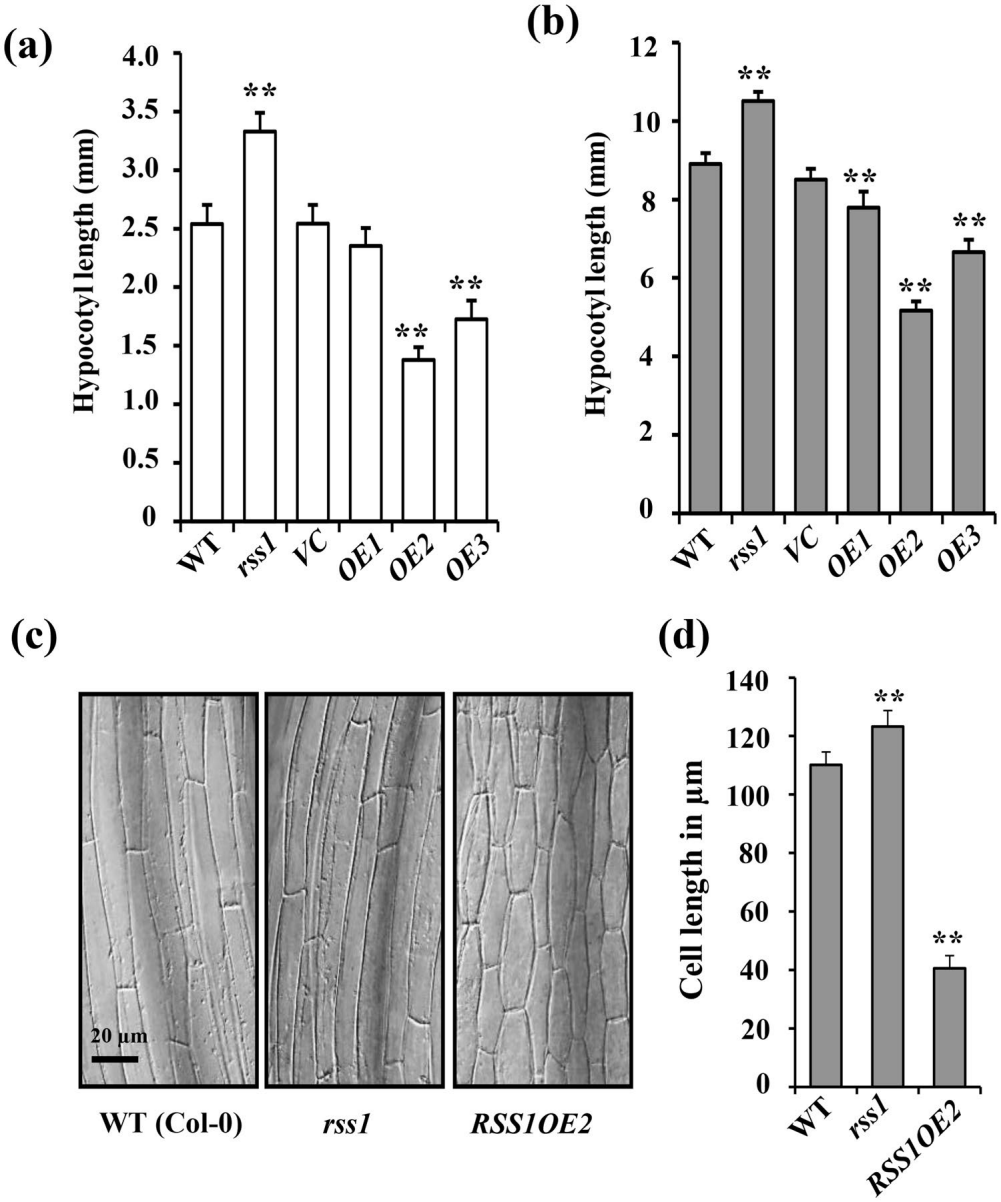
RSS1 is a negative regulator of hypocotyl elongation growth. Phenotype analysis of 7-d-old WT (Col-0), homozygous T-DNA insertion mutant *rss1* and transgenic overexpression lines (*OE1, OE2, OE3*) along with vector control (*VC*) seedlings growing under (**a**) long day conditions and (**b**) continuous darkness. The hypocotyls of *rss1* mutants were significantly longer than WT while *RSS1OE2* seedlings had relatively shorter hypocotyls. **(c)** Micrograph of hypocotyls of WT, *rss1* and *RSS1OE2* seedlings (Bars = 20 μm). (**d**) Quantification of hypocotyl epidermal cell length of WT, *rss1* and *RSS1OE*2seedlings. Values represent the average from two biological replicates each having 15 seedlings and error bars represent SE. (Student’s t-test; P < 0.01; **WT vs. mutant).

To functionally characterize and identify the functions of RSS1, T-DNA insertion mutant (*SALK*_*043980C*) was obtained from ABRC. Homozygous T-DNA insertion mutants were screened using PCR based genotyping (Fig. S6a). However, we observed two distinct phenotypes in seedlings of homozygous line (Fig. S6b). The *SALK*_*043980C* line obtained from ABRC was polymorphic and contained one or more T-DNA insertion elements i.e. *SALKseq_043980.0* & *SALK_043980.52.90.x* (*RSS1*); *SALKseq_043980.2* (*AT2G01750*) and *SALKseq_043980.201* (*AT4G014210*). We then again performed PCR based genotyping to eliminate associated polymorphisms and obtained a homozygous *rss1* T-DNA insertion line with no other polymorphism associated with it (Fig. S6c,d). To further clean the mutant from all possible background mutations and ensure a clean population the plant was further backcrossed two times with wild-type (Col-0). *RSS1* transcript level was significantly reduced in homozygous *rss1* T-DNA mutant as compared to the WT suggesting that the *rss1* is a knock-down line (Fig. S6e). *RSS1* overexpressing lines were also created in Arabidopsis. *RSS1* transcript levels were checked in three different homozygous overexpression lines. Line *OE2* showed very high RSS1 transcript accumulation (Fig. S6f). The difference in *RSS1* transcript accumulation in different over-expression lines reflected in their respective phenotype severity. The *rss1* knock-down mutant seedlings showed relatively longer hypocotyls and bigger cotyledons and on the other hand, transgenic overexpression seedlings had dark-green cotyledons and short hypocotyls as compared to WT and vector control (*VC*) plants, suggesting RSS1 to be a negative regulator of plant growth (Fig. 4a; Fig. S6g). Under etiolated conditions, *rss1* knock-down mutant showed marginally increased hypocotyl elongation while over-expression lines showed significantly smaller hypocotyl as compared to WT seedlings (Fig. 4b). Based on the phenotype and the extent of expression, overexpression line *RSS1OE2* was used for further studies. Microscopic analysis of light grown *rss1* mutant and *RSS1OE* seedling hypocotyls revealed that the *rss1* mutant has significantly longer epidermal cells as compared with WT, while the severe dwarfism of *RSS1OE* was due to reduced cell elongation (Fig. 4c,d).

### RSS1 integrates Glc and shade avoidance response

**Figure 5:**
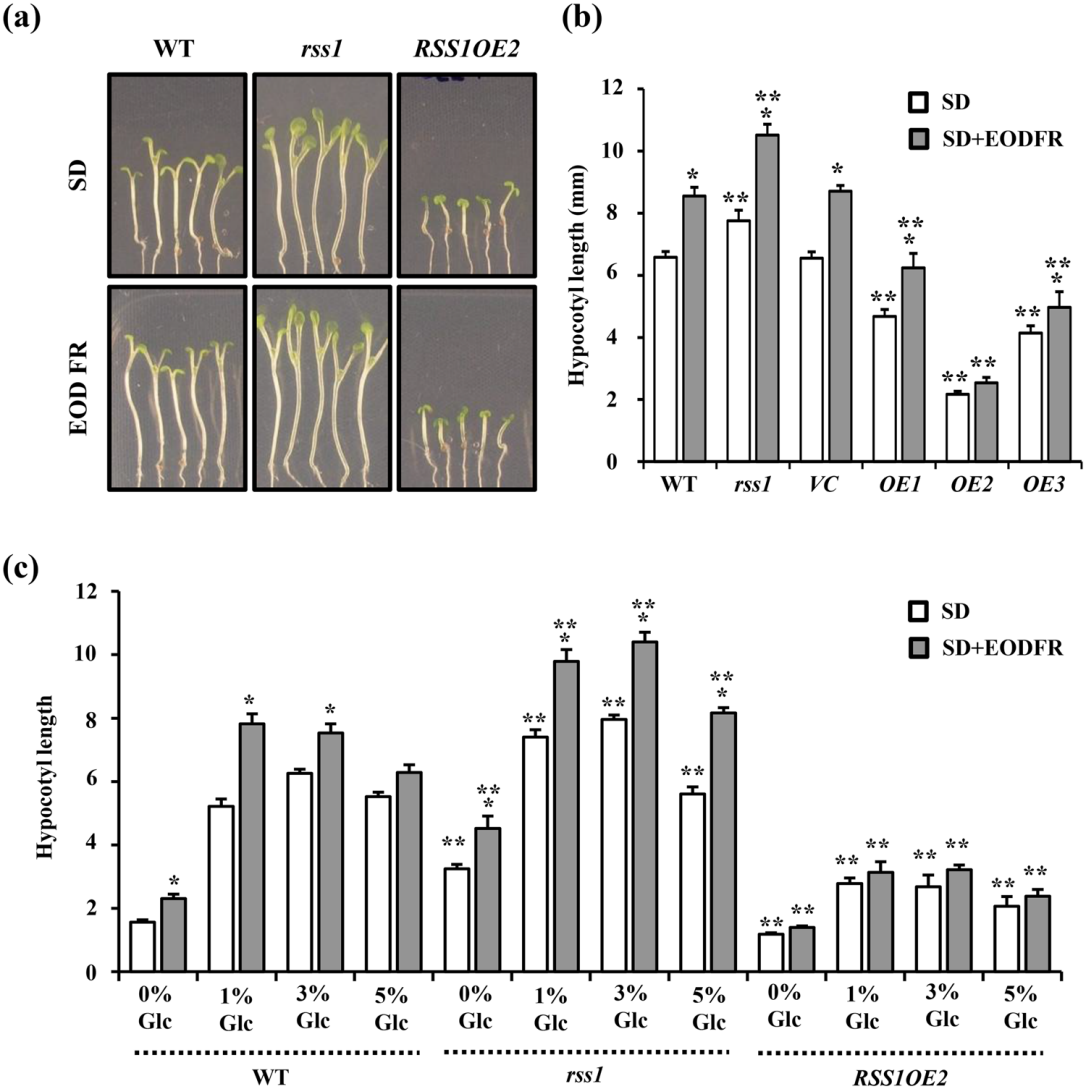
RSS1 is involved during Glc mediated regulation of EODFR-induced hypocotyl elongation growth. (**a**) Pictures and (**b**) hypocotyl length measurements of 6-d-old short day grown seedlings of WT (Col-0), *rss1* mutant, vector control (*VC*) and homozygous *RSS1OE2* lines after 1 h EODFR treatment. The *rss1* mutant showed significantly more hypocotyl elongation whereas *RSS1OE2* seedlings showed less response for shade induced hypocotyl elongation growth. The *RSS1OE2* seedlings were resistant for EODFR induced hypocotyl elongation. (**c**) The *RSS1OE2* seedlings were found to be less responsive for both Glc and EODFR in terms of hypocotyl elongation whereas the *rss1* mutant showed relatively more hypocotyl elongation upon EODFR treatment even in presence of higher Glc concentrations in the medium as compared to that of WT suggesting a probable negative role for RSS1 during Glc control of shade response. Values represent the average of three biological replicates each having 15 seedlings and error bar represents SE. (Student’s t-test; P < 0.05; *control vs. treatment; **WT vs. mutant).

Since *RSS1* encodes for a non-DNA binder atypical bHLH protein and its transcription is also induced upon simulated shade application^5^ (Fig. 3d), we checked the effect of EODFR treatment on hypocotyl elongation growth of WT, *rss1* and *RSS1OE* seedlings. The *rss1* knock-down lines show more hypocotyl elongation growth as compared with WT which was further increased upon EODFR treatment (Fig. 5a,b). The *OE* line showed resistance towards EODFR-induced hypocotyl elongation suggesting a negative role for RSS1 during shade avoidance response (Fig. 5a,b). The combined effect of Glc and simulated shade upon WT, *rss1* and *RSS1OE* line were analysed and it was observed that *rss1* showed more response for both Glc as well as EODFR induced hypocotyl elongation growth as compared to WT (Fig. 5c, Fig. S7a). Interestingly, *rss1* was able to show significant EODFR induced hypocotyl elongation growth at higher doses of Glc that were inhibitory for WT. On the other hand, *RSS1OE* line showed less sensitivity towards both Glc as well as EODFR induced hypocotyl elongation growth and even lower doses of Glc were not able to induce EODFR-mediated hypocotyl elongation in *RSS1OE* (Fig. 5c). In addition to simulated shade, 4-d-old light grown seedlings of WT, *rss1* and *RSS1OE* were also subjected to elevated temperature (29 °C) for 3d and hypocotyl elongation growth was quantitatively examined. The *rss1* seedlings showed more hypocotyl elongation growth whereas the RSS1*OE* line showed resistance towards high temperature-induced hypocotyl elongation as compared to WT (Fig. S7b). Taken together, our results suggested that Glc might utilize RSS1 mediated machinery to regulate hypocotyl elongation growth under different environmental conditions.

### RSS1 is involved in light regulated plant growth and development

**Figure 6:**
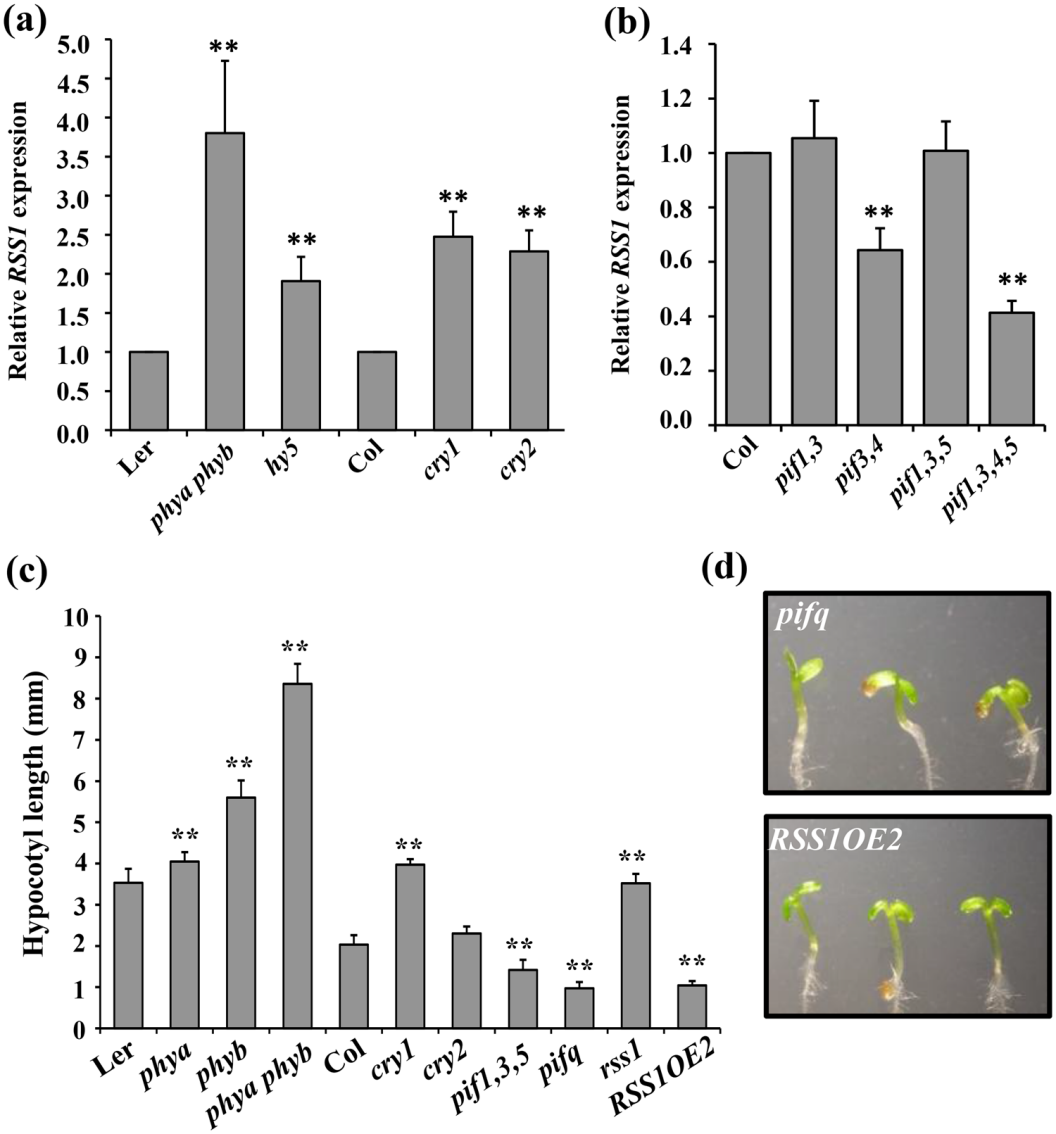
RSS1 is transcriptionally regulated through various light signaling components. (**a**) The relative *RSS1* transcript levels in light signaling mutants *phya phyb*, *hy5*, *cry1* and *cry2* and *hy5* as compared with their respective WTs. (**b**) Real-time PCR based analysis of PIFs mediated transcriptional control of *RSS1*. (**c**,**d**) Comparison of hypocotyl growth phenotypes in 7-d-old seedlings of WT and different light signaling mutants with that of *rss1* mutant and *RSS1OE2* seedlings. The *RSS1OE2* seedlings were phenotypically similar to the *pifq* mutant seedlings. Expression values represent the average of two biological replicates with three technical replicates each. Values for hypocotyl length are the average of two biological replicates each having 15 seedlings and error bar represents SE. (Student’s t-test; P < 0.05; **WT vs. mutant).

Light is one of the most important factors regulating plant growth and development, either by facilitating photosynthesis, or through photoreceptor mediated signaling that controls plant development. The light signal stimulates seedling de-etiolation/photomorphogenesis by inhibiting hypocotyl elongation followed by cotyledon expansion and chloroplast development. *RSS1*_*pro*_ *:GUS* expression pattern in seedlings subjected to continuous light and dark revealed that *RSS1* is repressed by light (Fig. S8a). However, this light-repression of *RSS1* can be an indirect effect and also be correlated with its repression with Glc which is a product of photosynthesis. We also compared the light-mediated inhibition of hypocotyl elongation growth of WT, *rss1* mutant and *RSS1OE* transgenic seedlings. The *rss1* mutant showed relatively less inhibition of hypocotyl elongation growth while the transgenic overexpression plants showed enhanced sensitivity to light and showed relatively more inhibition of hypocotyl growth at lower light flux (up to 1000 lux) as compared with that of WT (Fig. S8b). However, high light flux (7000 lux) could show similar inhibitory effect on hypocotyl elongation. To check if the components of light signaling pathway are involved in transcriptional regulation of *RSS1*, we tested basal *RSS1* transcript levels in light signaling defective mutants. *RSS1* transcript levels were significantly higher in different photoreceptor mutants such as *phya phyb* double mutant, *cryptochrome1* (*cry1*) and *cry2* mutants as compared to their respective WTs (Fig. 6a). *RSS1* has previously been shown to be a direct target of PIF4^50^. *RSS1* expression was also found to be significantly reduced in *pifq* mutant (Fig. 6b). We further compared the hypocotyl lengths of WT, *rss1*, *RSS1OE* seedlings with that of mutants defective in various light signaling components. The hypocotyl length and seedling phenotypes of *RSS1OE* line was similar to that of *pifq* **s**uggesting a role for *RSS1* in PIF mediated growth and development (Fig. 6c,d).

### RSS1 might act as a transcriptional co-repressor of HBI1 and BEE2

**Figure 7:**
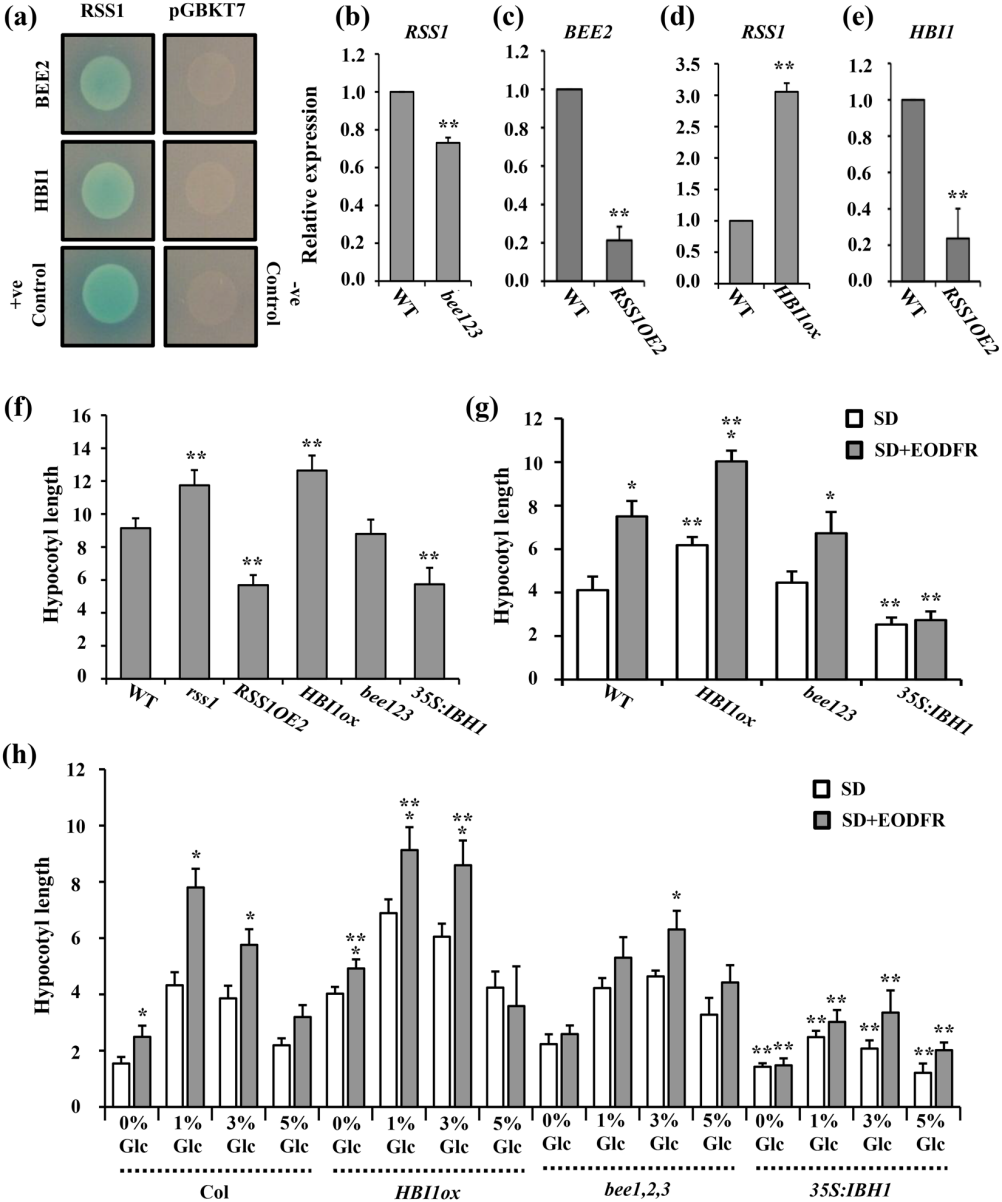
RSS1 interacts with known regulators of elongation growth. (**a**) Yeast two-hybrid assays of interactions between RSS1 and BEE2 or HBI1 proteins. (**b**) *RSS1* transcript levels were slightly reduced in *bee1*, *2*, *3* triple mutants. (**c**) RSS1 overexpression highly represses *BEE2* transcript abundance. (**d**,**e**) Effect of HBI1 overexpression on *RSS1* transcript abundance and *vice versa*. Overexpressing HBI1 caused induction of *RSS1* whereas *RSS1OE2* leads to *HBI1* transcript repression suggesting a negative interaction between both these proteins. (**f**) The hypocotyl elongation phenotypes of *rss1* mutant were similar to *35 s:HBI1:YFP* seedlings further confirming a negative correlation between RSS1 and HBI1 functions. The hypocotyl elongation phenotype of *RSS1OE2* seedlings was similar to that of *35 s:IBH1* seedlings suggesting that RSS1 might possess functional similarity to IBH1. Analysis of EODFR induced hypocotyl elongation growth response in WT, 3*5 s:HBI1:YFP*, *bee1,2,3* and *35 s:IBH1* seedlings growing on (**g**) ½ MS medium (1% Suc) and (**h**) ½ MS medium supplemented without or with different Glc concentrations (0%, 1%, 3%, 5% w/v). The HBI1 overexpressing mutants showed constitutively more hypocotyl elongation and increased sensitivity towards EODFR whereas the IBH1 overexpressing seedlings showed reduced hypocotyl elongation and resistance towards EODFR treatment. Expression values represent the average of two biological replicates with three technical replicates each. Physiological data shown is the average of two biological replicates each having atleast 15 seedlings; error bars represent SE; (Student’s t-test; P < 0.01; *control vs. treatment; **WT vs. mutant).

Under extended shade, activated PIFs induce the transcription of many non-DNA binding HLHs that can form a negative feedback loop and inhibit DNA binding activities of PIFs via heterodimerization. Besides PIFs, these different non-DNA binder HLHs could also inhibit other downstream DNA-binding bHLH factors such as BEE2 and HBI1. Both BEE2 and HBI1 are known regulators of hypocotyl elongation growth. We then investigated the probable involvement of RSS1 as a non-DNA binder transcriptional regulator (co-activator/repressor). Subcellular localization analysis using *35s*_*pro*_:*RSS1-YFP* translational fusion revealed RSS1 to be specifically localized to the nucleus, which was further confirmed by co-localization of *35s*_*pro*_:*RSS1-YFP* with DAPI (Fig. S9a). Protein-protein interaction analysis using yeast two-hybrid approach revealed that RSS1 could interact with both BEE2 and HBI1 (Fig. 7a). The *RSS1* transcript level was comparable to WT in *bee1bee2bee*3 mutant whereas *RSS1OE* line showed significantly reduced *BEE2* expression (Fig. 7b,c). The *35S:HBI1:YFP* line showed more *RSS1* transcript accumulation whereas the *HBI1* transcript levels were highly repressed in *RSS1OE* line (Fig. 7d,e). *RSS1* displays an opposite gene expression pattern to that of *BEE2* and *HBI1* under one or more developmental stages and/or treatment conditions (Fig. S9b). Under both normal as well as simulated shade conditions, hypocotyl elongation phenotype of *rss1* mutant was comparable to that of *HBI1OE* line (Fig. 7f,g; Fig. S10). We used another transcriptional co-repressor belonging to non-DNA binder HLH category, IBH1 as a control in our experiments. The *IBH1* overexpression line showed essentially the similar hypocotyl elongation phenotypes as *RSS1OE* line under both normal as well as shaded conditions (Fig. 7f,g; Fig. S10). The *HBI1OE* line showed more EODFR induced hypocotyl elongation at all Glc concentrations similar to *rss1* mutant (Fig. 7h; Fig. S10). The *IBH1OE* line, on the other hand, showed resistance to EODFR-induced hypocotyl elongation and Glc was also not able to exert any effect on these seedlings (Fig. 7h; Fig. S10). These results revealed a general mode for Glc-mediated regulation of shade response that might involve the tripartite bHLH/HLH module.

### RSS1 might affect auxin response machinery to regulate elongation growth

**Figure 8:**
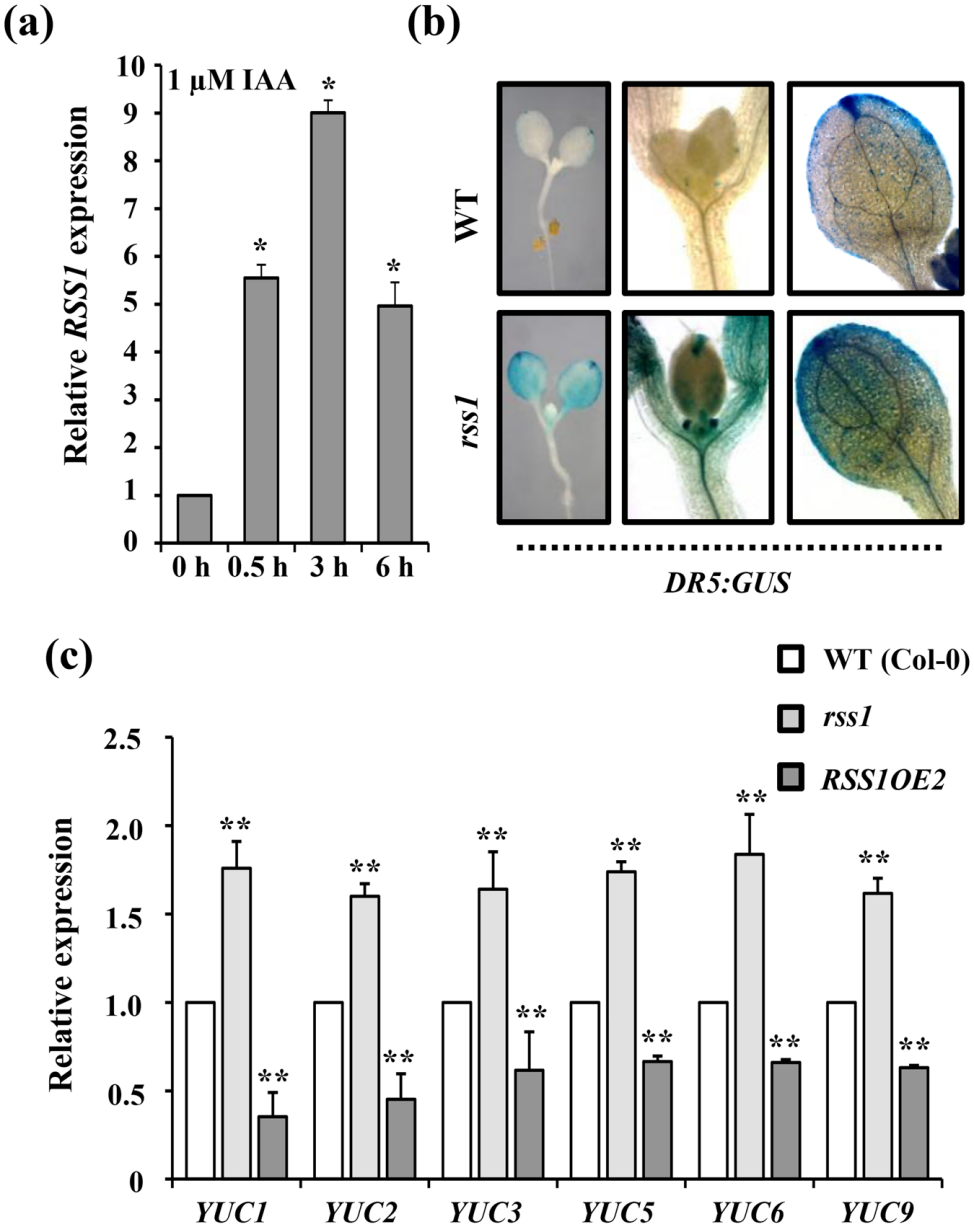
RSS1 might negatively affect auxin biosynthesis/signaling in Arabidopsis. (**a**) Effect of IAA treatment on *RSS1* transcript abundance. *RSS1* is an early auxin inducible gene as its transcript levels are significantly induced 30 min. post IAA treatment. (**b**) Analysis of *DR5:GUS* reporter expression in the *rss1* mutant background. (**c**) Basal transcript levels of auxin biosynthesis related genes *YUC1, YUC2, YUC3, YUC5, YUC6* and *YUC9* in WT (Col-0), *rss1* mutant and *RSS1OE2* seedlings. The *DR5:GUS* reporter expression and basal transcript levels of *YUC* genes are significantly induced in the *rss1* mutant background suggesting a negative role of RSS1 in auxin biosynthesis and activity. Expression values represent the average of two biological replicates with three technical replicates each and error bars represent SE. (Student’s t-test; P < 0.05; *control vs. treatment; **WT vs. mutant).

In our previous study, RSS1 transcript levels were found to be significantly induced by 3 h auxin treatment^37^. To check the kinetics of auxin mediated transcriptional regulation of *RSS1*, 5d old WT Arabidopsis seedlings were treated with 1 µM IAA for 30 min, 3 h and 6 h. The expression of *RSS1* was found to be significantly enhanced within 30 min of IAA treatment suggesting it to be an early auxin inducible gene (Fig. 8a). The local auxin dynamics defines the magnitude, locale and timing of various developmental responses and organogenesis in plants. Auxin biosynthesis and spatial accumulation of newly synthesized auxin in hypocotyl epidermal cells is responsible for hypocotyl elongation growth under shaded conditions. The *rss1* mutant showed phenotypes previously reported to be typical of elevated auxin level^55^, such as elongated hypocotyl, expanded leaves and increased rosette size as compared to the wild type (Fig. S11a), To analyze the effect of RSS1 on auxin homeostasis and/or activity *in planta*, we introduced auxin-responsive *DR5:GUS* reporter construct into the *rss1* mutant background via genetic crossing. The *DR5:GUS* expression was highly induced in cotyledons as well as hypocotyls in *rss1* background as compared with WT (Fig. 8b). We also checked the free IAA accumulation in cotyledon as well as hypocotyls of WT, *rss1* mutant and *RSS1OE2* seedling using immunolocalization assay and the *rss1* mutant showed more auxin accumulation while the *RSS1OE2* line showed significantly reduced auxin accumulation as compared to wild type (Fig. S11b). Similarly, the transcript levels of *YUCCA* gene family members were also significantly enhanced in *rss1* mutant and repressed in *RSS1OE* line as compared to WT (Fig. 8c). Taken together our results suggest that RSS1 might be negatively affecting auxin homeostasis and/or activity to regulate hypocotyl elongation growth under various signals.

**Figure 9:**
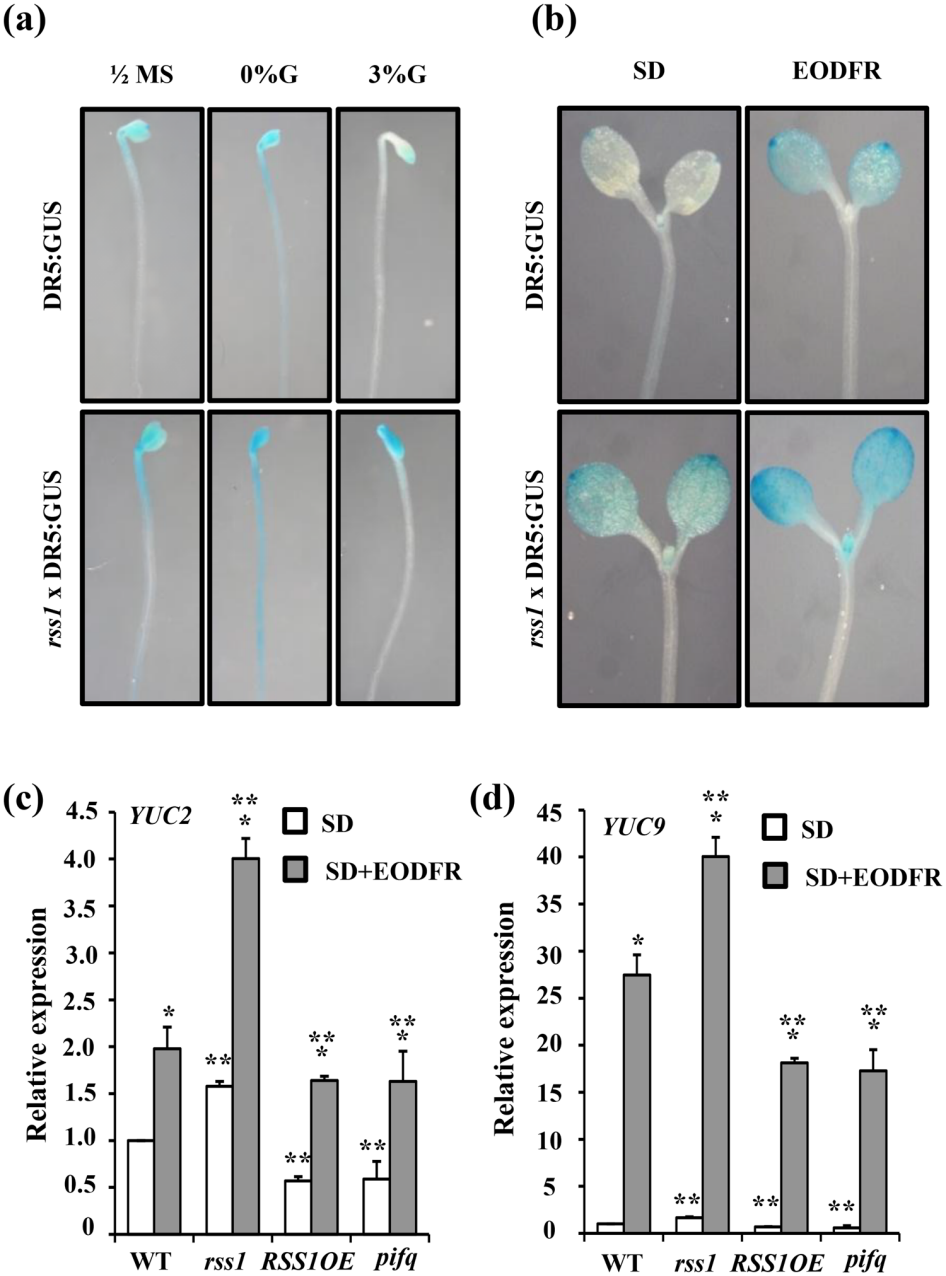
RSS1 integrates Glc, light/shade and auxin signals in Arabidopsis. (**a**) The Glc mediated inhibition of *DR5:GUS* reporter expression is significantly reduced in the *rss1* mutant background. (**b**) The EODFR mediated induction of *DR5:GUS* reporter expression is more in the *rss1* mutant background. Comparison of simulated shade (EODFR) mediated induction of (**c**) *YUC2* and (**d**) *YUC9* transcript levels in WT (Col-0), *rss1*mutant, *RSS1OE2* and *pifq* mutant seedlings. EODFR treatment caused more induction in *YUC2* and *YUC9* transcripts in *rss1* mutants whereas the shade-mediated induction of these genes was relatively less in *RSS1OE2* line and *pifq* mutant seedlings as compared with WT. Expression values represent the average of two biological replicates with three technical replicates each and error bars represent SE. (Student’s t-test; P < 0.05; *control vs. treatment; **WT vs. mutant).

**Figure 10:**
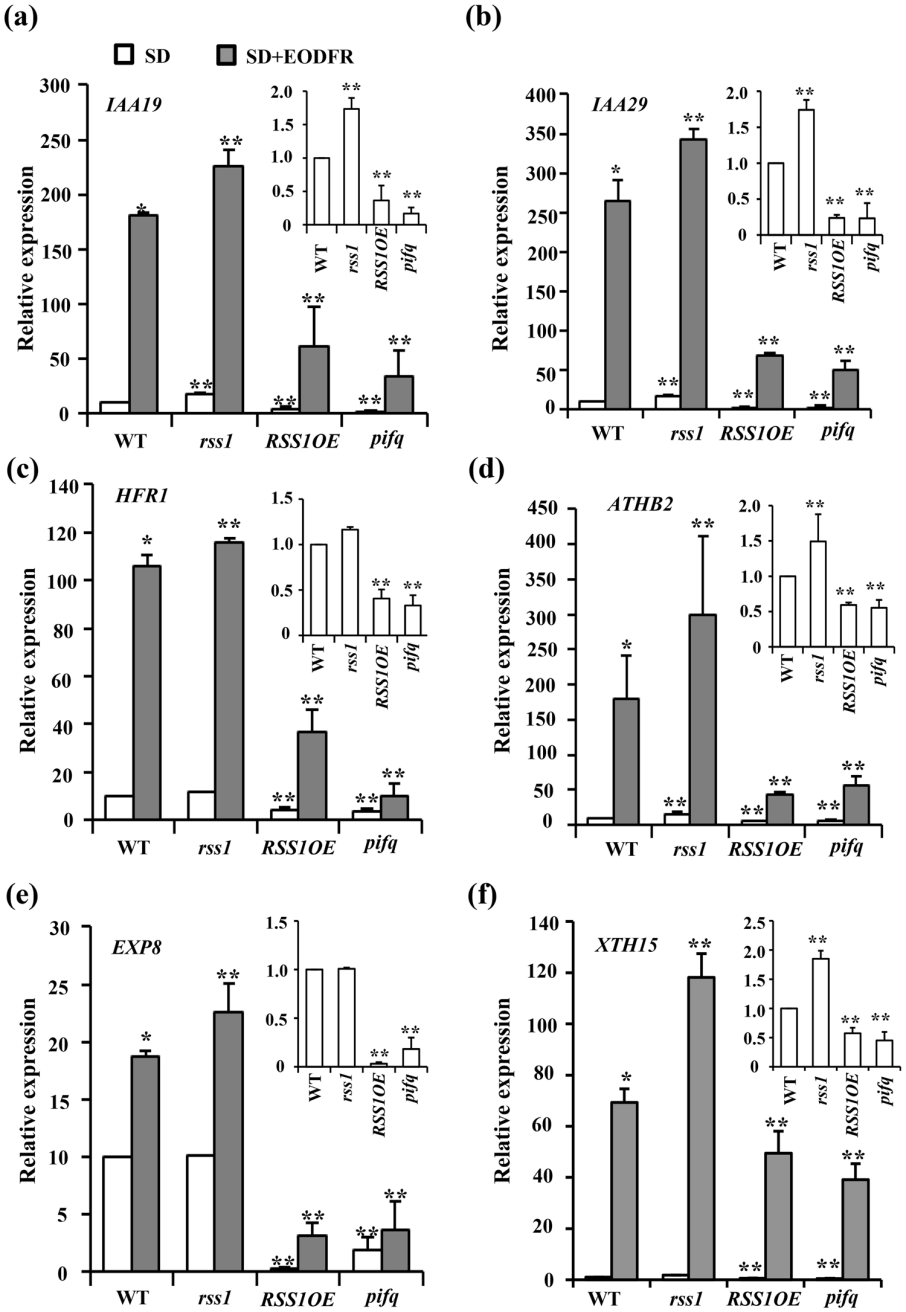
RSS1 affects transcriptional regulation of PIF targets/shade responsive genes in Arabidopsis. Comparison of simulated shade (EODFR) mediated induction of transcript levels of selected shade responsive genes that are also well-known targets of PIF4/HBI1 such as (**a**) *IAA19*; (**b**) *IAA20*; (**c**) *HFR1*; (**d**) *ATHB2*; (**e**) *EXP8* and (**f**) *XTH15* in WT (Col-0), *rss1* mutant and *RSS1OE2* transgenic lines. The basal transcript levels in *rss1, RSS1OE2* and *pifq* seedlings relative to the WT are given in inset. EODFR treatment caused more induction in the transcript levels of selected shade responsive genes in *rss1* whereas the shade-mediated induction of these genes was highly reduced in *RSS1OE2* line and *pifq* mutant seedlings as compared to the WT. Expression values represent the average of two biological replicates with three technical replicates each and error bars represent SE. (Student’s t-test; P < 0.05; *control vs. treatment; **WT vs. mutant).

Further, the Glc mediated inhibition of *DR5:GUS* expression was lost and the EODFR mediated induction of *DR5:GUS* expression was significantly enhanced in *rss1* mutant background as compared to WT background (Fig. 9a,b). During shade avoidance response, PIFs promote transcription of the *YUCCA* genes involved in auxin biosynthesis. Apart from PIFs, HBI1 can also activate many genes involved in cell elongation growth and auxin biosynthesis. To obtain further insight into the role of RSS1 in shade avoidance response, the relative expression of selected shade responsive PIF4/HBI1 target genes was studied in WT, *rss1* mutant, *RSS1OE* transgenic lines and *pifq* mutant seedlings under both normal as well as EODFR conditions. *YUC2*, *YUC9*, *IAA19*, *IAA29*, *ATHB2*, *HFR1*, and cell elongation promoting genes *EXP8* and *XTH15* were selected as they have been previously shown to be rapidly induced on exposure to shade^16–18,56^. Basal transcript levels of these genes were significantly reduced in both *RSS1OE* transgenic and *pifq* mutant as compared to WT while *rss1* showed slightly more transcript accumulation. Also, the simulated shade (EODFR) mediated induction of these shade responsive genes was perturbed in *RSS1OE* line similar to the *pifq* mutant (Figs 9c,d and 10) suggesting a negative correlation between the activity of RSS1 and PIF transcription factors. Taken together our results suggest that RSS1 might inhibit PIF/HBI1 activity at the molecular level. These results suggest that Glc might use RSS1 and other HLH factors as a switch to control elongation growth under various environmental conditions.

## Discussion

Plants being immobile, cannot escape the adversities of their surroundings. However, plants possess exuberant plasticity to modulate their growth in accordance to ambient environmental cues. This developmental plasticity largely depends upon cellular elongation. In Arabidopsis seedlings, different light quality and availability correlates with distinct hypocotyl growth phenotypes. Sugars/Glc as an energy molecule and signaling molecule can affect hypocotyl elongation growth^43,48,49,57^. We have previously shown that Glc signaling could regulate hypocotyl elongation growth in both light grown as well as etiolated seedlings of Arabidopsis by affecting a phytohormonal signaling cascade^43^. In this study, we show that exogenous Glc alone (3% Glc vs 0% Glc w/v^37^); could regulate 55% of the genes previously reported to be differentially regulated by simulated shade (3 h FR/WL)^5^. Majority of this overlapping transcriptome (75%) is antagonistically regulated by both these signals (Fig. S2). Besides simulated shade, Glc could also regulate transcriptome overlapping with that influenced by phytohormones regulating cellular elongation such as auxin and BR^37,39,43^. Further, Glc alone could regulate transcript accumulation of approximately one-third (~35%) of the genomic targets of central growth regulatory B-A-P (BZR1-ARF6-PIF4) circuit which are also the primary transcription factors of BR, auxin and light signaling pathways, respectively (Fig. S3). These results suggest a key role for sugars in coordinating cellular elongation growth according to the environmental conditions and hormonal status of the plant. Exogenous Glc treatment could also affect EODFR-induced hypocotyl elongation in a dose dependent manner (Fig. 1). An intact HXK1-dependent signaling pathway is needed for optimal Glc response under shaded conditions (Fig. 1). Interestingly, Glc was not able to affect hypocotyl elongation upon EODFR treatment in light signaling defective mutants suggesting dependence of sugar signaling upon light signaling associated with shade avoidance response (Fig. 2). Our attempt to identify molecular nodes of integration between light, Glc and phytohormonal signals has lead us to the identification of a candidate gene with unknown biological function (*AT3G29370; RSS1*). The *RSS1* transcript levels were repressed by Glc and induced by simulated shade (Figs S4, S5, S6 Fig. 3). The HXK1 dependent pathway seems to be the prominent mode for Glc regulation of *RSS1* transcription (Fig. 3). Molecular and functional characterization of RSS1 was pursued to understand the molecular mechanism of integration between light, Glc and phytohormone signaling network in regulating hypocotyl elongation. The role of *RSS1* in early seedling growth and development was studied using knock-down and transgenic overexpression lines. RSS1 can modulate hypocotyl elongation under various growth conditions. Overexpression of RSS1 suppressed cell elongation, while the *rss1* knock-down mutant showed slight but significant increase in cell elongation (Fig. 4). Transgenic plants overexpressing RSS1 had shorter hypocotyls whereas *rss1* knock-down seedlings exhibited more hypocotyl elongation in darkness as well as in long day conditions as compared to WT. The *RSS1pro:GUS* expression were also repressed in continuous light conditions compared to dark (Fig. S8a). Further, the light-mediated inhibition of hypocotyl elongation was delayed in *rss1* mutant (Fig. S8b). RSS1 was also found to be involved in shade-avoidance responses and response to high temperature (Fig. 5; Fig. S7). The simulated shade mediated induction of *RSS1* transcript as well as hypocotyl elongation growth responses of *rss1* mutant and *RSS1OE2* seedling under EODFR conditions suggested that RSS1 acts as a negative regulator of shade-avoidance response. Also, the Glc mediated inhibition of shade-induced hypocotyl elongation was suppressed in *rss1* knock-down line suggesting that increasing doses of Glc can negatively regulate shade-induced hypocotyl elongation by controlling *RSS1* activity either transcriptionally or post-transcriptionally (Fig. 5).

*RSS1* encodes for a small nuclear localized HLH protein and does not have a DNA binding domain similar to other atypical bHLH factors such as PAR1, PAR2 and IBH1. In plants, around 26% bHLH proteins are classified as atypical bHLH factors due to lack of a functional basic domain required for DNA-binding^52,54^. These atypical bHLH factors might act as both transcriptional co-suppressors by forming heterodimers with bHLH factors or indirect activators of bHLH factors by interacting with a non-DNA binder HLH factor^12,18,52,54,58,59^. The tri-antagonistic HLH/bHLH module forms a second tier of interacting transcription factors downstream of the central growth regulatory circuit consisting of the B-A-P module^14^. PIF4 is an integral factor of B-A-P module and acts as negative regulators of photomorphogenesis and promote shade-avoidance^4^. In optimal light conditions, activated phytochromes target PIFs for degradation thereby regulating its activity. Many studies have demonstrated that the non-DNA binder atypical HLH factors work downstream of many environmental and hormonal signals and play fundamental roles in regulating cell elongation and plant growth^18,50,60–63^. This complex network integrates multiple environmental as well as endogenous signals, such as light, temperature and phytohormone to regulate the transcriptome.

Being a non-DNA-binding HLH protein similar to PAR1, PAR2 and IBH1, RSS1 might also be involved in regulation of transcription and act as a negative regulator of other bHLH proteins. Hypocotyl elongation phenotype and gene expression studies suggested that RSS1 functions downstream to PIF4 to regulate hypocotyl elongation growth (Fig. 6). As *RSS1* transcription is under control of PIF4, and *RSS1OE* has similar phenotypic defects as *pifq* mutant in response to simulated shade and high temperature, RSS1 might work via inhibiting PIF4 activity through feedback regulation to inhibit hypocotyl elongation growth in shade. Protein-protein interaction analysis using yeast two-hybrid screens has identified HBI1 and BEE2 as interactor proteins of RSS1 (Fig. 7). Both BEE2 and HBI1 belong to subfamily 18 of bHLH proteins and regulate cell elongation under multiple external and endogenous signals^18^. Since the expression pattern of *RSS1* was found to be opposite to both HBI1 as well as BEE2 at various developmental stages, it suggested that RSS1 could act as a negative regulator for these interactors for one or more developmental processes.

The *RSS1* transcript levels were induced by auxin. The transcript abundance analysis of YUC family of genes as well as *in situ* IAA accumulation analysis in *rss1* mutant and *RSS1OE* seedlings along with the analysis of auxin responsive *DR5:GUS* reporter expression in *rss1* background further confirmed that RSS1 might be involved in negative regulation of auxin biosynthesis (Fig. 8, Fig. S11). Also, Glc mediated inhibition of *DR5:GUS* expression was lost in *rss1* mutant background while the EODFR-mediated induction of *DR5:GUS* expression was significantly enhanced in *rss1* mutant background suggesting RSS1 to be acting at the junction of Glc-, light/shade- and auxin-signaling interaction network involved in cellular elongation (Fig. 9). Further, transcript analysis of few shade-responsive candidate genes, which are genomic targets of both PIF4 and HBI1, such as *YUC2*, *YUC9*, *IAA19*, *IAA29*, *ATHB2*, *HFR1*, *EXP8* and *XTH15* revealed that RSS1 might inhibit transcriptional activation of these genes by preventing DNA binding ability of HBI1 to its downstream target genes (Figs 9 and 10).

Our study provides important evidence that places sugar/Glc signaling amongst multiple phytohormones as well as environmental signaling pathways regulating the transcriptome for cell elongation. The triple *bee123* mutant showed WT-like sensitivity while the *35 s:HBI1-YFP* line showed enhanced hypocotyl elongation growth similar to *rss1* mutant under both normal as well as simulated shade conditions and Glc application. Interestingly, the *35 s*:*IBH1* line also showed resistance for both Glc as well as simulated shade induced hypocotyl elongation similar to *RSS1OE* line. These results suggest additional HLH factors may also contribute to Glc mediated regulation of elongation growth under shade conditions. Increasing doses of Glc (3% Glc) could repress the transcript levels of various HLH factors, such as PAR1, IBH1 and HFR1; and could also inhibit the transcription of bHLH factors, such as PIF4, HBI1, BEE1, 2, 3 (Table S3). Integrating previous studies as well as results of our physiological and molecular analysis, we present a hypothetical model of Glc mediated regulation of elongation growth and RSS1 action downstream to multiple phytohormone and environmental signals including shade (Fig. S12). We hypothesise that either a threshold transcript level is needed for *RSS1* to perform its function as a negative regulator or there might be some other post transcriptional mechanisms regulating *RSS1* function eventually regulating the final signal output during hypocotyl elongation growth. We propose that changing sugar/Glc levels and cellular energy status might be involved in maintaining the balance between various bHLH/HLH factors through both transcriptional as well as post-translational modes that ultimately optimize cellular elongation growth under different growth conditions. Whether Glc could regulate the activities of these HLH and/or bHLH factors at post-translational levels remains to be investigated. Further dissection of the effect of Glc on protein–protein and protein–DNA interactions dynamics, both spatially as well as temporally will be important for building a better understanding of signaling network involved in regulation of hypocotyl elongation and seedling fitness under changing environmental conditions.

## Methods

### Plant materials and growth conditions

Arabidopsis (*Arabidopsis thaliana*) ecotypes Col-0, Ler, and En-2 were used as wild-type controls. Seeds of *rss1* (AT3G29370, SALK_043980 C), *gin2-1* (AT4G29130, CS6383), *rgs1-1* (AT3G26090, CS6537), *gpa1-4* (AT2G26300, CS6534) and *thf1-1* (AT2G20890) were obtained from the Arabidopsis Biological Resource Center (http://www.arabidopsis.org/abrc/). The following lines were obtained from the original published sources: *35S:HBI1-YFP* [AT2G18300^18^], *AtIBH1-Ox* [At2g43060^60^], *bee123* [AT1G18400, AT4G36540, AT1G73830^64^]. Seeds were surface sterilized and imbibed at 4 °C in dark for 48 h. Imbibed seeds were germinated and grown vertically on ½ MS medium (1% Suc; 0.8% Agar w/v) in a climate-controlled growth room (22 °C ± 2 °C, 60 µmol m^−2^ s^−1^ light intensity, 16:8 light/dark cycle) for 5 d. Etiolated growth was studied as described previously. For End-Of-the Day Far-Red/EODFR treatment, seedlings were grown in short day (8:16 light/dark cycle, 60 µmol m^−2^ s^−1^ light intensity) conditions and exposed to FR for 1 h (15 µM m^−2^ s^−1^) at the end of the light cycle for 3-4 days. The different light sources were provided by LEDs (SNAP-LITE Quantum devices, inc). The chemicals and treatment concentrations were used as described previously^41,43^. The EODFR treatment has been frequently used in past to induces the shade avoidance response^2,5,51,65,66^.

### Measurement of Hypocotyl Elongation Growth

All end-point analyses were performed on day 7. Seedlings were photographed using a Nikon Coolpix digital camera. Hypocotyl length was measured using the ImageJ program (http://rsb.info.nih.gov/ij/) from National Institutes of Health.

### Gene Expression Analysis

Total RNAs were extracted using RNeasy Plant Mini Kit (Qiagen). cDNAs were synthesized from 2 µg of total RNA using the high-capacity cDNA Archive kit (Applied Biosystems). Real-time quantitative polymerase chain reaction (qPCR) assays were performed with the ABI 7900HT Fast Real-Time PCR System (Applied Biosystems) using the SYBR Green PCR master Mixture (Applied Biosystems). Assays were performed with at least two biological replicates having three technical replicates each, and the relative transcript levels were normalized to the expression level of the internal control 18S ribosomal RNA gene. The mRNA levels for each candidate gene in different samples were determined using the delta delta cycle threshold method^67^. Primers for real-time PCR were designed for all the genes preferentially from the 3′ end of the gene using Primer Express version 2.0 (PE Applied Biosystems) with default parameters. All primers used for this assay are listed in Fig. S13.

### Phylogenetic analysis

For multiple sequence alignment, AT3G29370 protein and other bHLH protein sequences were retrieved from TAIR and aligned using Clustal omega^68^. Phylogenetic analyses and tree construction was performed using MEGA6^69^ according to the neighbour-joining method. The bootstrap values were calculated after 1000 retrials.

### Identification of homozygous T-DNA insertion mutant and generation of transgenic Arabidopsis lines

To identify homozygous *rss1* T-DNA insertion lines, genomic DNA was isolated from plant leaves and subjected to PCR genotyping using specific set of primers. The homozygous T-DNA insertion line was backcrossed with WT (Col-0) to further eliminate the background mutations; the homozygous line obtained from segregating F_2_ population was used for further studies. To find the exact position of T-DNA insertion in the gene, the amplified PCR product was purified and sequenced. Full-length CDS of RSS1 with stop codon were amplified from Arabidopsis genomic DNA and cloned into pENTRY vector (Invitrogen), sequence verified, and recombined into pEarleyGate100^70^ vector using Gateway LR Clonase Enzyme Mix (Invitrogen). The *RSS1* promoter was amplified from the genomic DNA by PCR starting from 2000 bp upstream of the start codon using specific primers and cloned into pENTRY vector (Invitrogen) and recombined into pMDC164^71^ using Gateway LR Clonase Enzyme Mix (Invitrogen). These constructs were then introduced into Arabidopsis WT plants via the floral dip method^72^. All primers used in this study are listed in Fig. S10.

### Crossing Arabidopsis plants and screening of double mutants

*DR5:GUS* reporter in *rss1* mutant background was generated by making genetic cross between healthy plants of homozygous *rss1* T-DNA insertion mutant and *DR5:GUS* reporter line. The F_1_ generation was allowed to self-pollinate. Homozygous *rss1* T-DNA insertion was confirmed using PCR based genotyping and *DR5:GUS* reporter was confirmed using GUS histochemical staining in segregating F_2_ population. The primer sequences used for genotyping have been included in Fig. S13.

### GUS Histochemical Staining

GUS activities in *RSS1*_*pro*_ *:GUS*, *DR5:GUS* and *rss1xDR5:GUS* lines under various growth and treatment conditions were determined using a standard GUS histochemical staining procedure as described^43^. Seedlings after treatment were subsequently incubated at 37 °C in a GUS staining solution [0.1 M sodium phosphate buffer, pH 7, 0.5 mM K3Fe(CN)6, 0.5 mM K4Fe(CN)6, 50 mM EDTA, and 1 mg mL21 5-bromo-4-chloro-3-indolyl-b-glucuronic acid] for 30 min to 40 min. The seedlings were then kept in 70% (v/v) ethanol for the removal of chlorophyll. The staining in the seedlings was then observed under Microscopy was done on Zeiss Axio Imager2 microscope using differential interference contrast (DIC) optics (Carl Zeiss, Germany) or on Nikon SMZ1500 Stereo-Zoom microscope, and photographs were taken with a Nikon Coolpix digital camera connected to a Nikon SMZ1500 Stereo-Zoom microscope. The experiment was repeated thrice, with each replicate having at least 10 seedlings, yielding similar results.

### Yeast Two-Hybrid Assay

Yeast two-hybrid assay was conducted using Matchmaker Gold Yeast two-hybrid System (Clontech, Mountain View, CA) according to manufacturer’s protocol. The CDS of *RSS1* was cloned in pGBKT7g through Gateway technology^73^ and used as a bait to screen normalized Mate & Plate Universal Arabidopsis Yeast two-hybrid cDNA library (Clontech, Mountain View, CA). The interaction of HBI1 and BEE2 was confirmed by cloning them in pGDAT7g and one-to-one interaction check with RSS1. pGBKT7-53 and pGADT7-T were used as positive control and pGBKT7-Lam and pGADT7-T were used as negative control for the experiments. The primers used for cloning are shown in Fig. S10.

### Immunolocalization of IAA

For free IAA accumulation estimation, 7 days old seedlings of WT (Col-0), *rss1* and *RSS1OE* were kept in histochoice clearing agent (sigma) for 20 min followed by washing twice with 100% ethanol for 5 min each. Tissues/samples were rehydrated by passing through gradients of ethanol (95%, 80%, 60%, 30% and sterile water) for 5 minutes each and followed by PBS wash; (2 × 10 minutes). Whole seedling *IAA* immunolocalization was performed using monoclonal anti-auxin antibody (A0855, sigma) as described previously^74^. Calorimetric detection was performed using NBT/BCIP based detection solution (Roche Diagnostics, India), as per company’s manual. Once visible signal was observed, seedlings were washed with PBS (2 × 10 minutes) followed by dehydration increasing gradients of ethanol (5 min each) and kept in histochoice clearing agent for 10 min. Seedlings were mounted on glass slides with 10% glycerol and imaged using a Nikon Coolpix digital camera connected to a Nikon SMZ1500 Stereo-Zoom microscope.

### Statistical analyses

All experiments reported in this study were performed at least three times yielding similar results. All values reported in this work are averages from at least two independent biological replicates each having at least 15 seedlings otherwise specified. Error bars represent standard error (SE). For all experiments, statistical differences between control and treatment conditions were analyzed using Student’s T-test evaluation with paired two-tailed distribution. P value cutoff was taken at P < 0.01 except where stated otherwise.

## Electronic supplementary material

Figure S1 to S13

Table S1

Table S2

Table S3
