# Supplementary material for: Arabidopsis RSS1 Mediates Cross-Talk Between Glucose and Light Signaling During Hypocotyl Elongation Growth
Source: Sci Rep. 2017 Nov 23;7:16101. doi: 10.1038/s41598-017-16239-y (PMC5701026; doi:10.1038/s41598-017-16239-y)
Supplement: Supplementary file 1 — Figure S1 to S13 [file 41598_2017_16239_MOESM1_ESM.pdf]

Arabidopsis RSS1 mediates cross-talk between Glucose and light signaling during hypocotyl elongation growth.

Manjul Singh[1,2,ψ], Aditi Gupta[1,2,ψ], Dhriti Singh[1], Jitendra P Khurana[2] and Ashverya Laxmi[1,*]

Supplementary Material

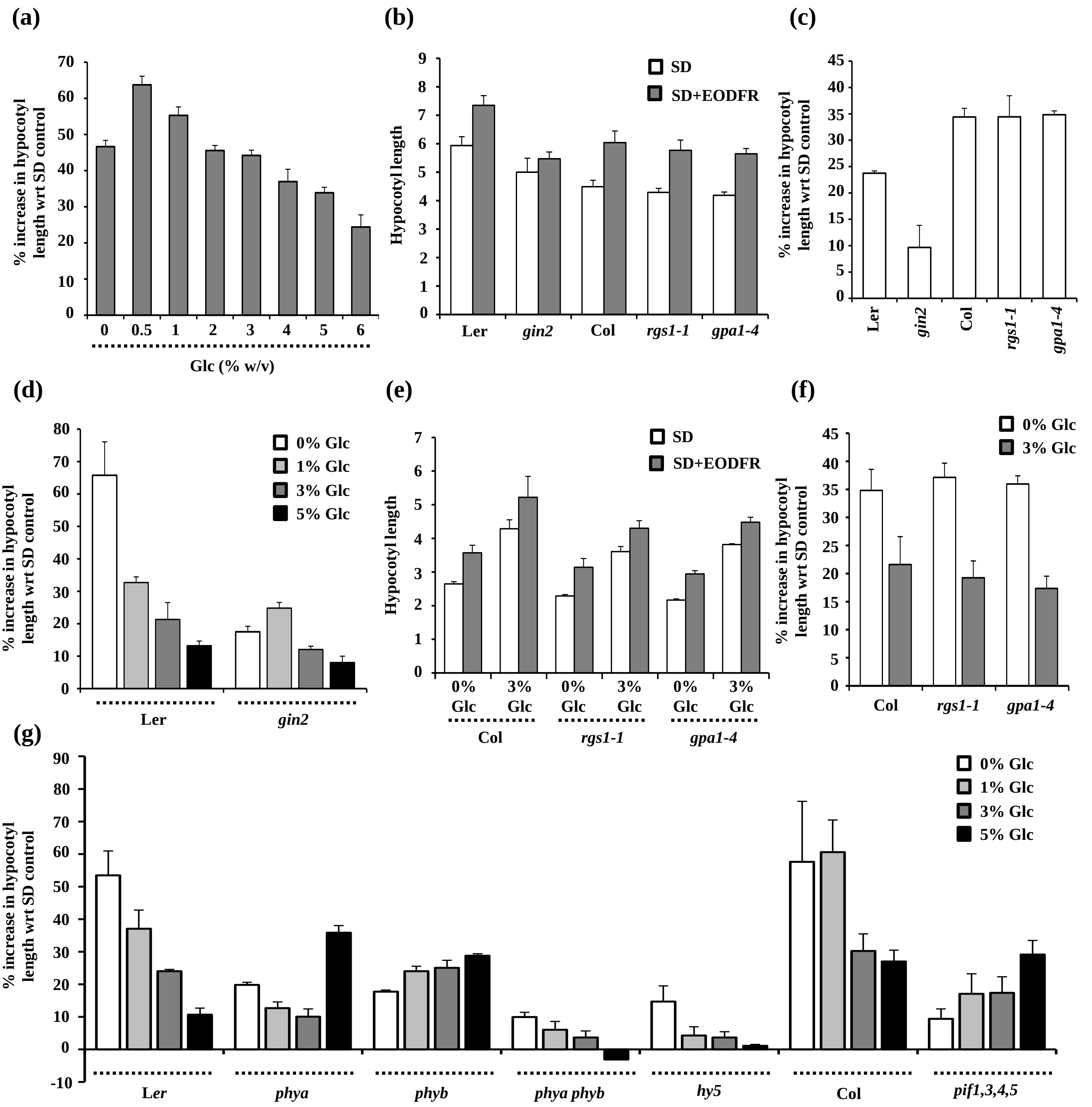

Figure S1. Effect of Glc in regulating simulated shade induced hypocotyl elongation growth.
(a, c, d, f, g) Graphs showing effect of EODFR treatment as percentage increase in hypocotyl length with respect to SD control. **(b)** The effect of EODFR on hypocotyl elongation growth of HXK1-dependent Glc signaling mutant *gin2* and HXK1-independent Glc signaling mutants *rgs1-1* and *gpa1-4* seedlings growing on **(b)** ½ MS medium and **(e)** ½ MS medium supplemented without or with Glc (0%, 3% Glc w/v). The HXK1-independent mutant *rgs1-1* and *gpa1-4* showed WT like sensitivity for EODFR induced hypocotyl elongation.

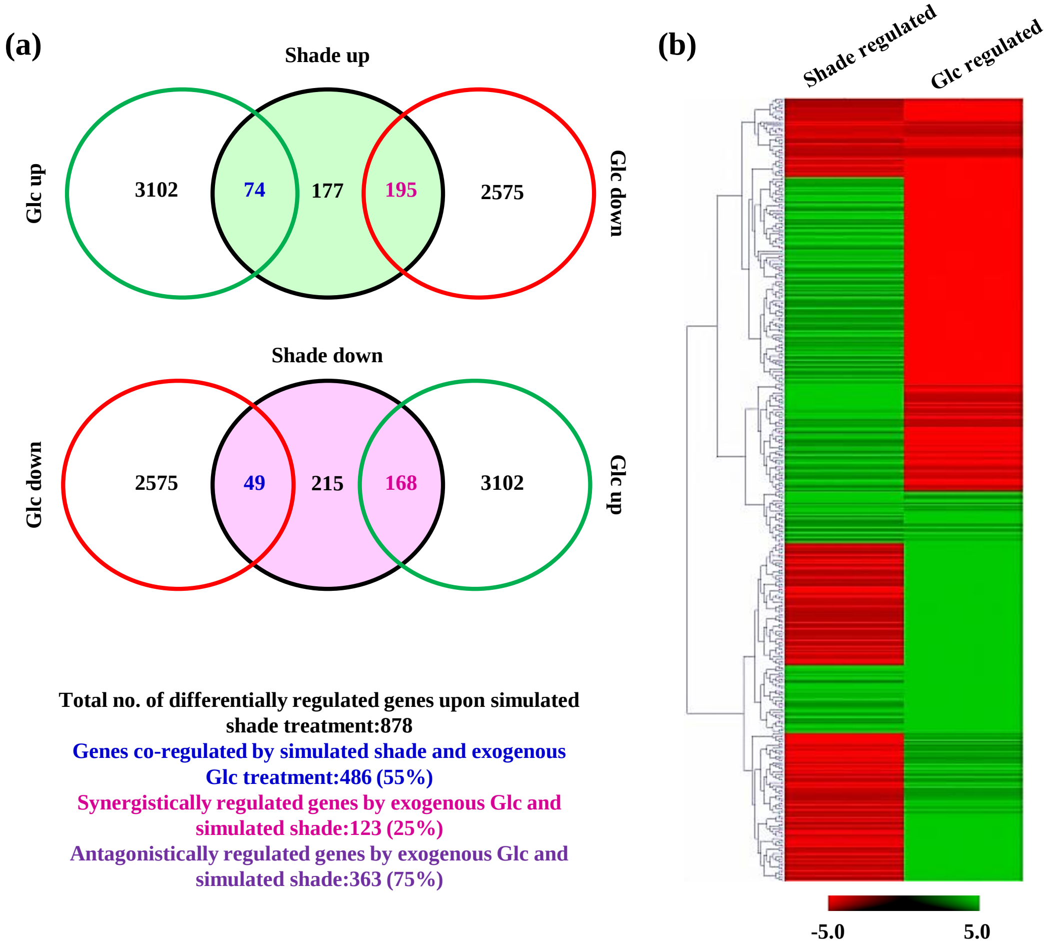

Figure S2. Glc mediated transcriptional regulation of simulated shade regulated genes. **(a)** Venn diagrams showing significant overlap between genes transcriptionally regulated by Glc (3% Glc vs 0% Glc) with genes differentially regulated by simulated shade (3h FR vs WL). **(b)** Hierarchical clustering of Glc and shade co-regulated genes. Glc could regulate approximately 55% of shade regulated transcripts; 75% of which are regulated in an antagonistic manner. The simulated shade regulated genes were taken from Leivar *et al.*, 2012 and overlapped with Glc regulated genes from Mishra *et al.*, 2009.

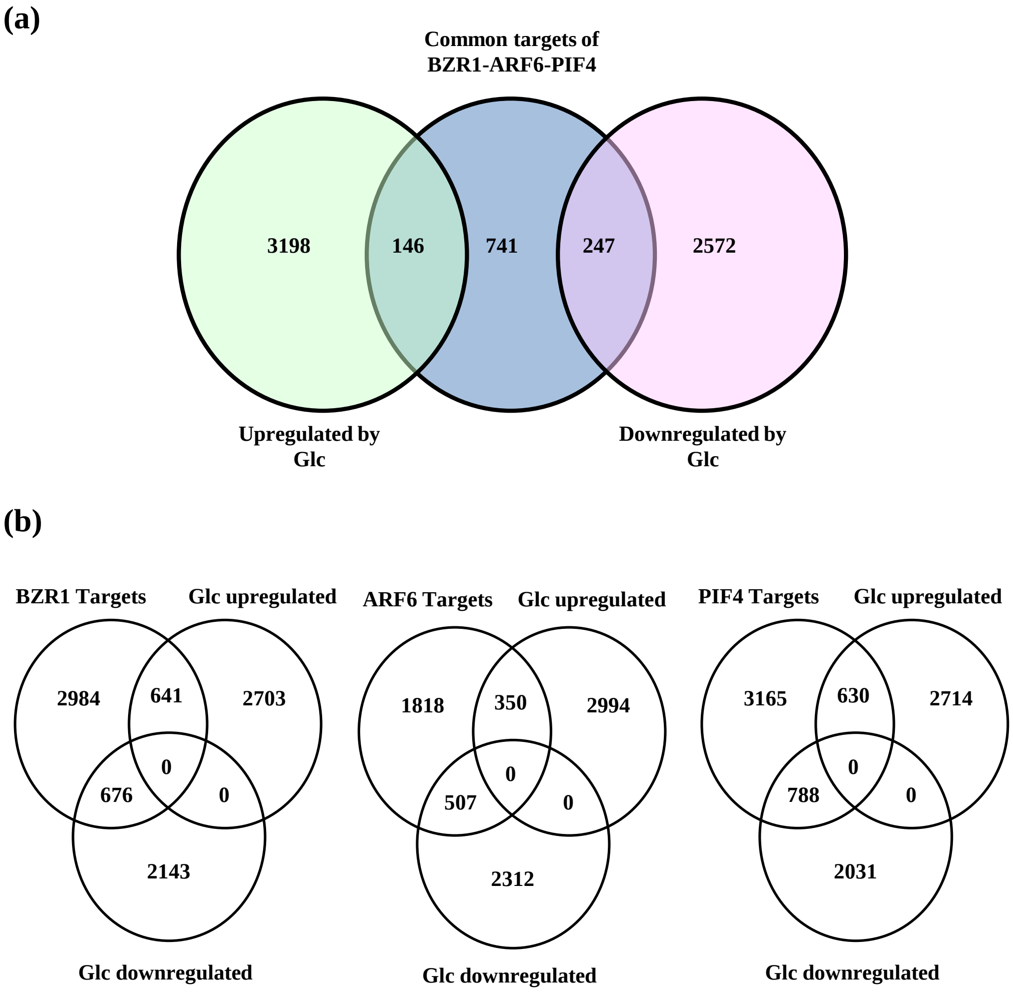

Figure S3. Glc mediated transcriptional regulation of BZR1-ARF6-PIF4 targets.
Venn diagram showing significant overlap between genes transcriptionally regulated by Glc with **(a)** common and **(b)** individual binding targets of BZR1, ARF6 and PIF4 respectively. The BZR1/ARF6/PIF4 binding targets were taken from Oh et al., 2014 and overlapped with Glc regulated genes light grown seedlings of Arabidopsis (Mishra *et al.*, 2009). Glc could regulate 35% of common targets of BZR1-ARF6-PIF4; 63% of which are repressed by Glc and only 37% were induced by Glc transcriptionally.

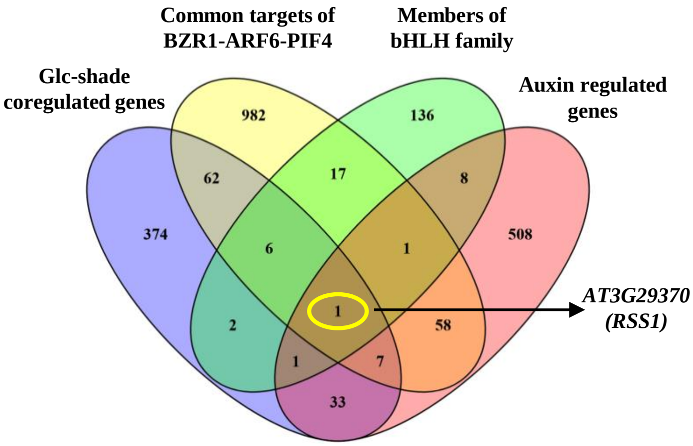

Figure S4. Venn diagram showing *RSS1 (At3G29370)* to be the only Glc-Shade-Auxin coregulated bHLH factor that is also a common target of BZR1-ARF6-PIF4 transcription factors regulatory module.

(a)

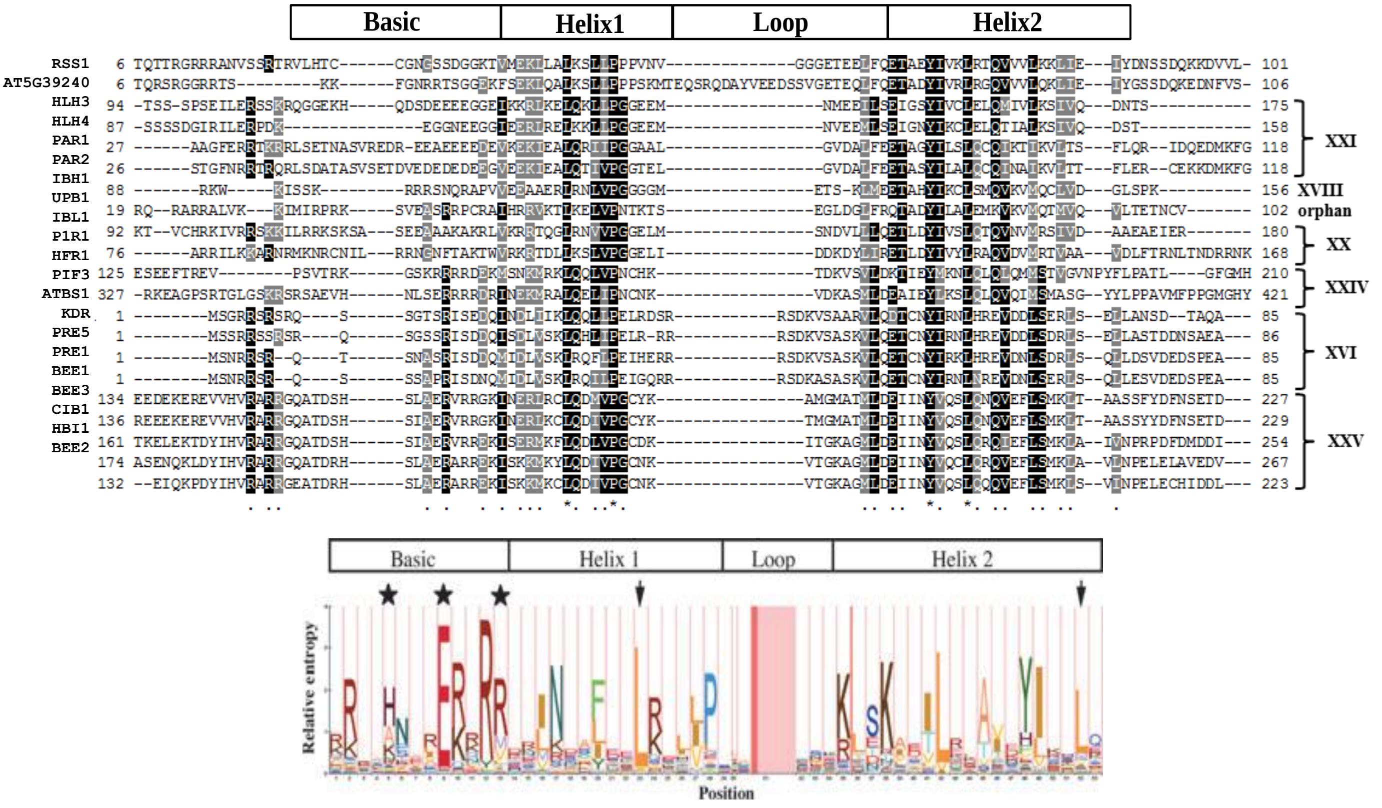

(b)

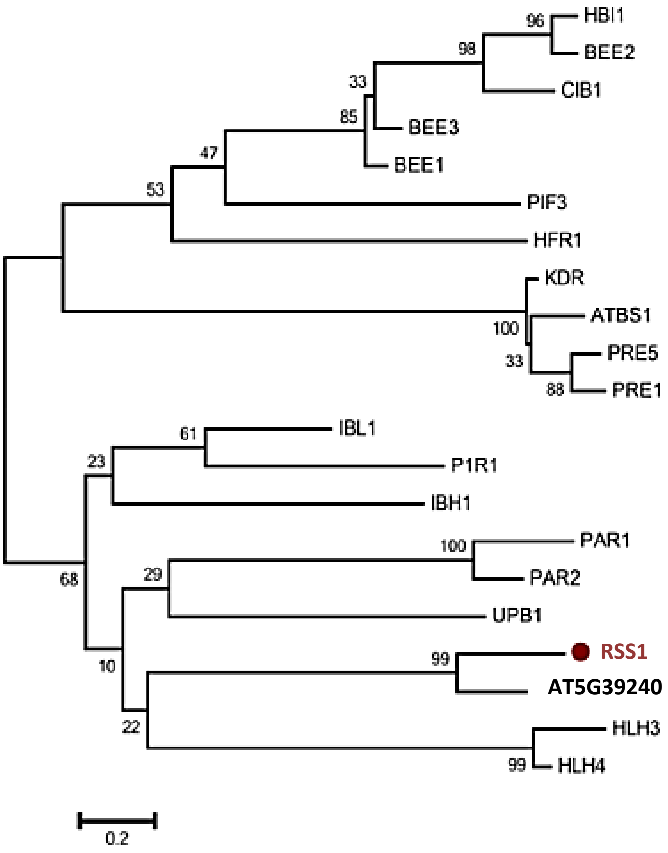

Figure S5. *In silico* analyses of RSS1.

(a) Multiple sequence alignment of bHLH domains from Arabidopsis from closest group proteins (Carretero-Paulet *et al.*, 2010). Identical residues are boxed in black. Gray boxes mark partially conserved residues. The position of the basic, helix and loop regions is indicated. Shading was performed by Box shade server. **(b)** Phylogenetic analyses were conducted using MEGA6 (Tamura *et al.*, 2013). Full-length protein sequences were aligned with Clustal omega (Sievers *et al.*, 2011), and the phylogenetic tree was constructed according to the neighbor-joining method. The bootstrap values out of 1000 retrials are indicated at each branch. The scale bar represents the number of substitutions per site.

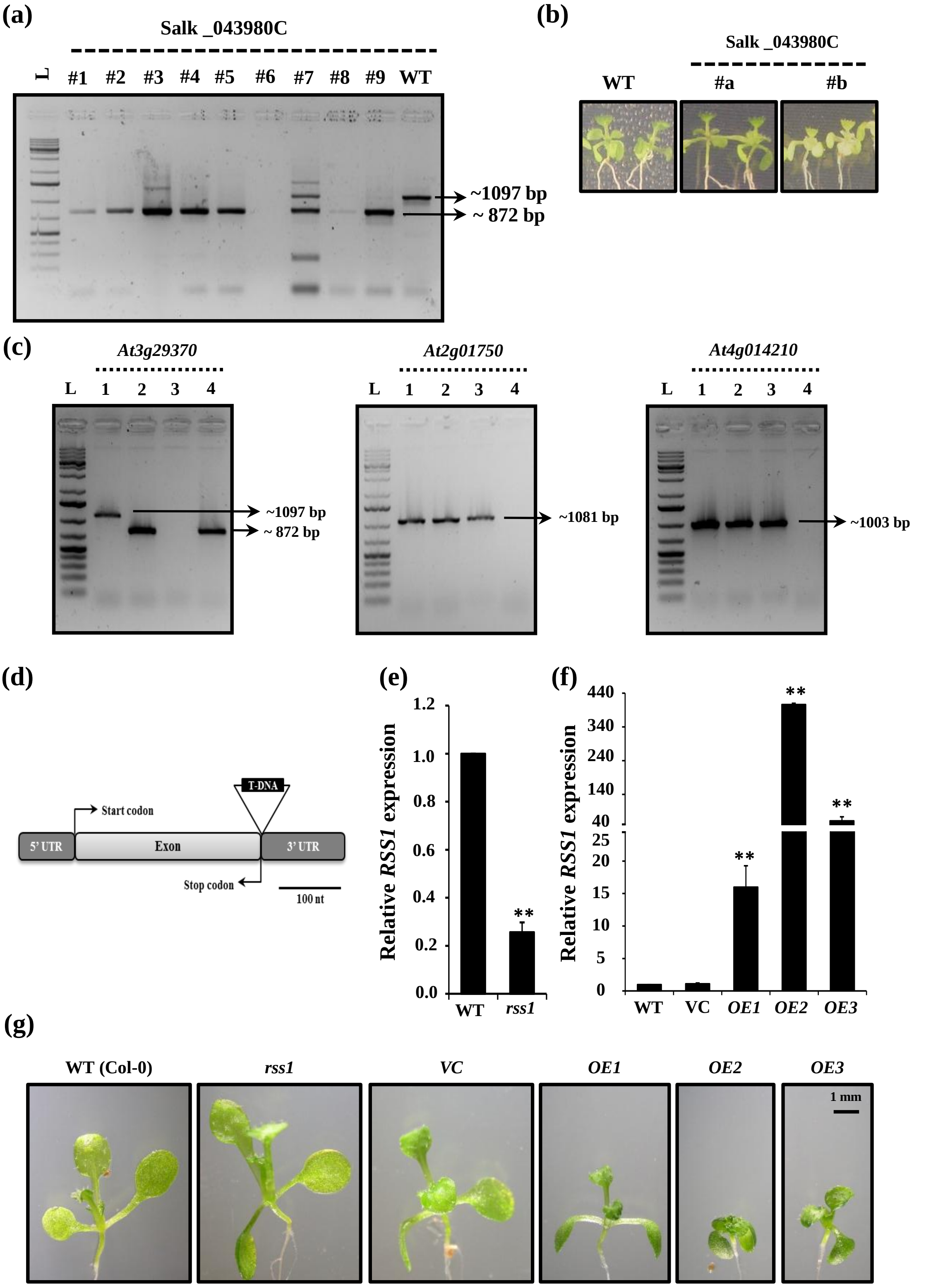

Figure S6 Screening and phenotype analysis of homozygous T-DNA insertion mutant and transgenic overexpression line of RSS1.

(a) PCR based genotyping to identify homozygous T-DNA insertion line (SALK_043980C). **(b)** Pictures showing two different phenotypes in the homozygous Salk line. **(c)** Elimination of other polymorphisms in the identified homozygous T-DNA lines. [L: 1 kb plus DNA Ladder; 1: WT (LP+RP); 2: Salk (LP+RP+LBb1.3); 3: Salk (LP+RP); 4: Salk (LBb1.3+RP)]. **(d)** Insertion position of T-DNA in homozygous *rss1* knockdown line. Real-time PCR analysis of *RSS1* expression in **(e)** homozygous knock-down line and **(f)** homozygous over-expression lines. **(g)** Hypocotyl growth phenotypes of 10-d-old light grown seedlings of WT (Col), homozygous rss*1* knockdown line and RSS1over expression lines (*OE1*, *OE2* and *OE3*).

Expression values represent the average of two biological replicates with three technical replicates each and error bar represents SE. (Student's T-test; P<0.05; ** WT vs. mutant).

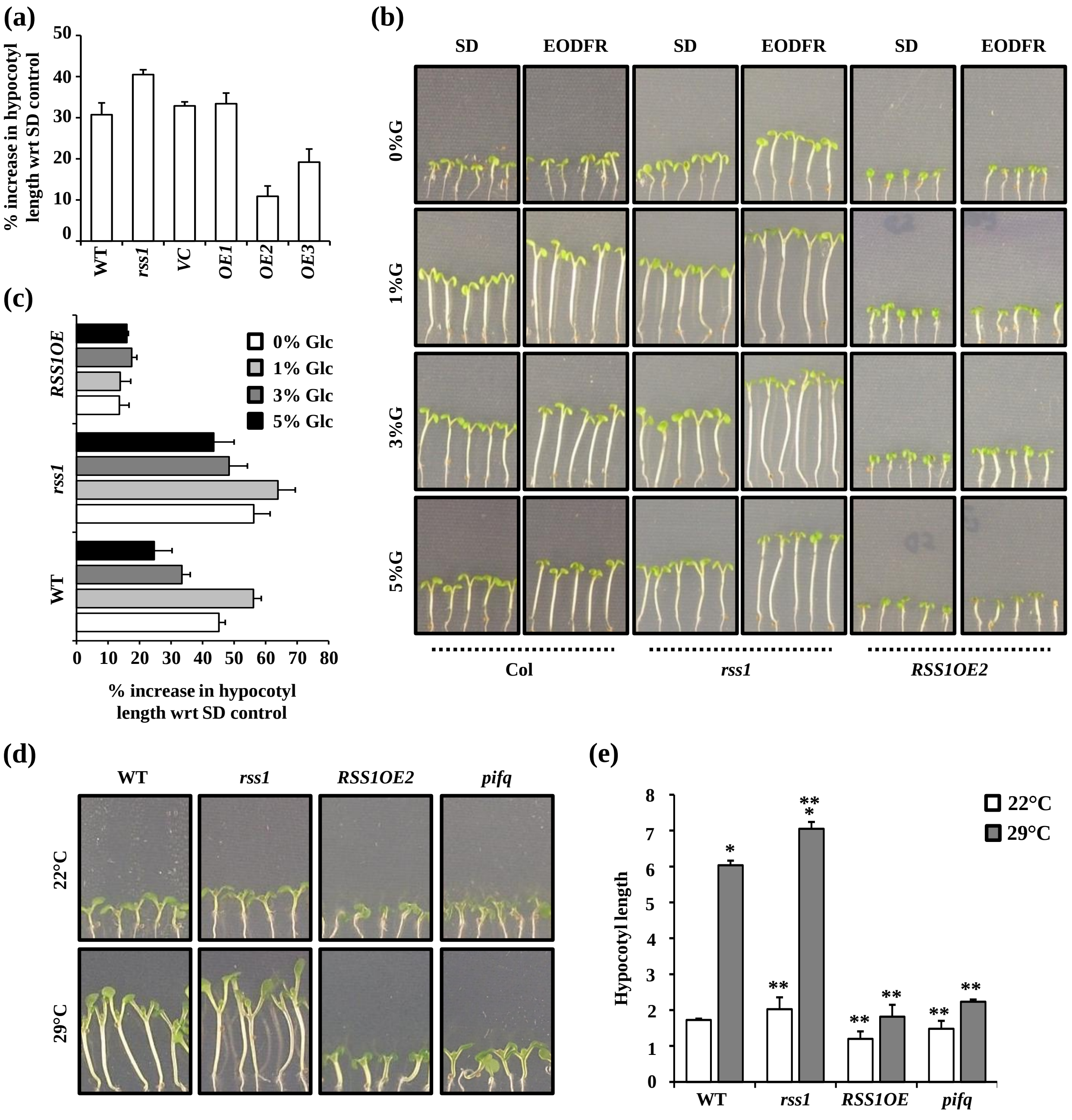

Figure S7: RSS1 inhibits Glc, shade and high temperature induced hypocotyl elongation growth.
(a) Graphs showing percentage increase in hypocotyl length of WT, VC, *rss1* mutant and *RSS1* over expression lines upon EODFR treatment as compared to SD control. **(b)** Pictures showing hypocotyl elongation of WT, *rss1*, and *RSS1OE2* seedling on Glc and EODFR treated conditions. **(c)** Percentage increase in hypocotyl length with respect to SD control on Glc and EODFR treated conditions in WT, *rss1* and *RSS1OE2*. **(d)** Pictures and **(e)** graphs comparing hypocotyl elongation growth phenotypes of 7d old seedlings of WT, *rss1*, *RSS1OE2* and *pifq* seedlings upon exposure to elevated temperature (29°C).

(a)

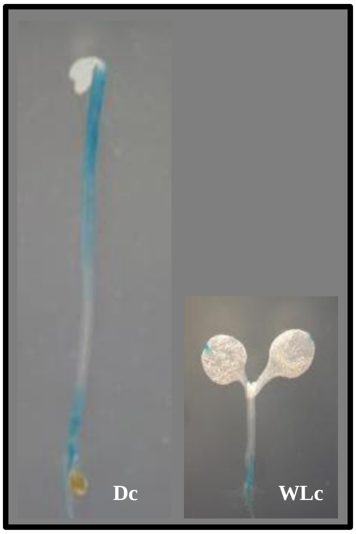

7-d-old *RSS1pro:GUS* seedlings

(b)

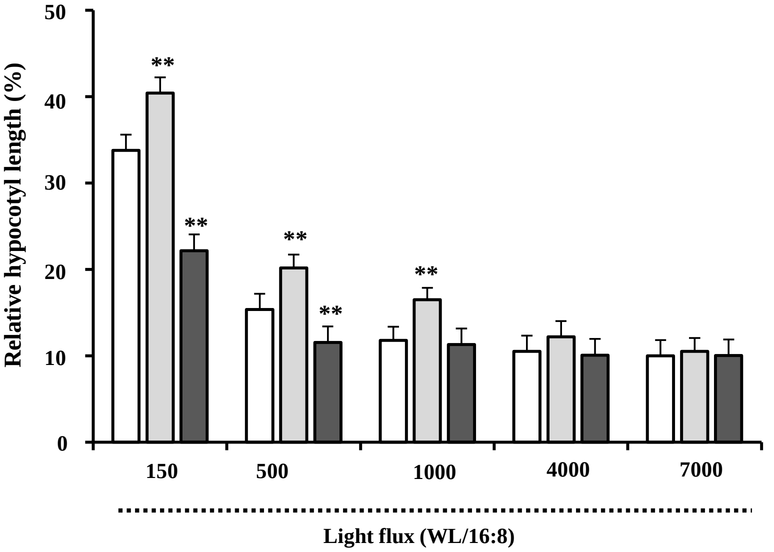

Figure S8. Light-mediated regulation of *RSS1* in Arabidopsis.

(a) *RSS1pro:GUS* expression in 7d old seedlings growing in dark and continuous white light (WLc) conditions. **(b)** Quantification of relative hypocotyl lengths (percentage of length in the darkness) of 7-d-old seedlings of WT, *rss1* mutant and *RSS1OE* plants grown under different light intensities.

Data shown is the average of two biological replicates and error bar represents SE. (Student's t-test; P<0.05; * control vs. treatment; ** WT vs. mutant).

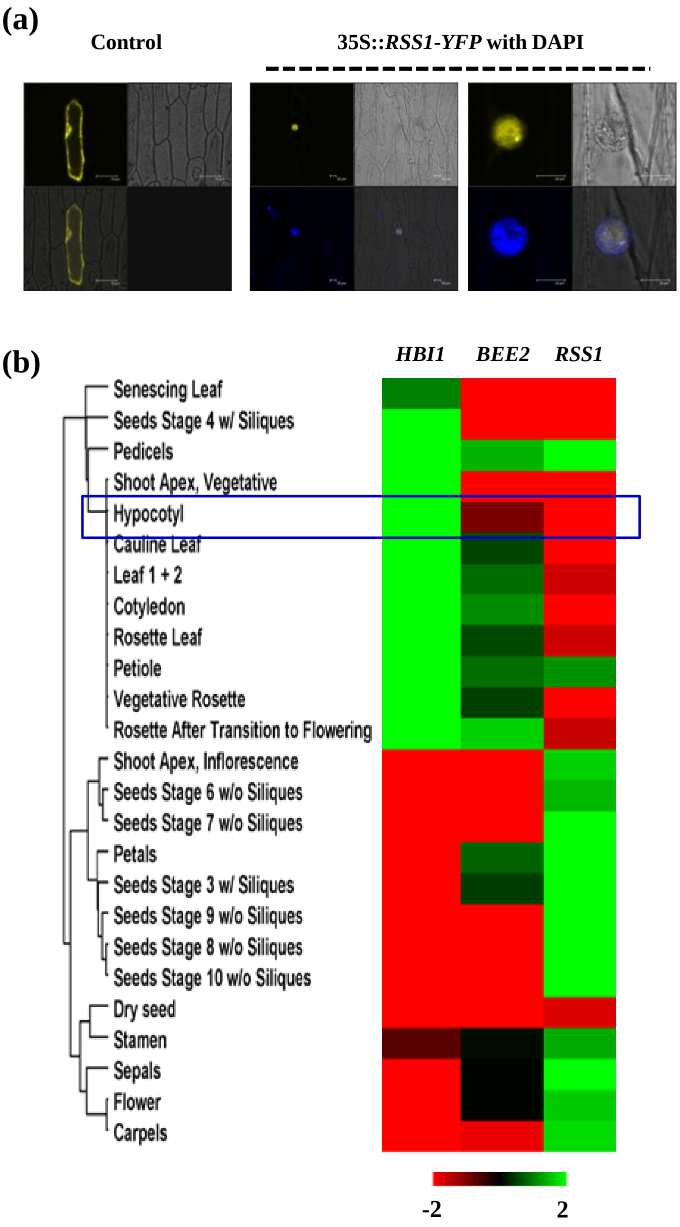

Figure S9. RSS1 is a nuclear localized transcriptional co-regulator.
(a) Subcellular localization analysis of RSS1 in onion epidermal cells. Co-localization of $35S_{pro}$:RSS1-YFP expression with DAPI confirmed the nuclear localization of RSS1. **(b)** Hierarchical clustering of *RSS1*, *HBI1* and *BEE2* expression profiles under various developmental processes. The bottom bar shows the color scale for regulation ratio relative to the control.

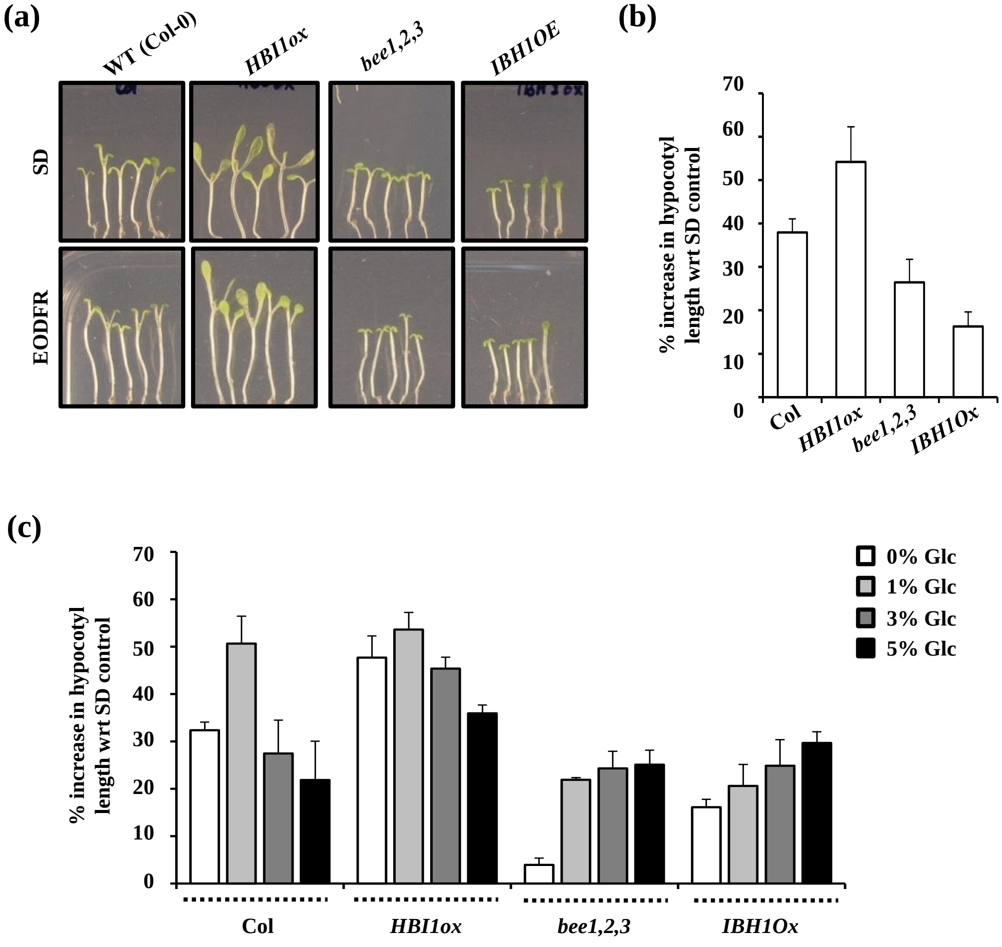

Figure S10. (**a)** Pictures and **(b)** graphs showing EODFR mediated promotion of hypocotyl elongation growth in WT (Col-0), *HBI1OE* line, *bee1,2,3* mutant and *IBH1OE* seedlings as compared with SD control. **(c)** Graphs showing percentage increase in hypocotyl length upon EODFR treatment with respect to SD control in WT (Col-0), *HBI1OE*, *bee1,2,3* mutant and *IBH1OE* seedlings growing on ½ MS medium supplemented with increasing concentration of Glc**.**

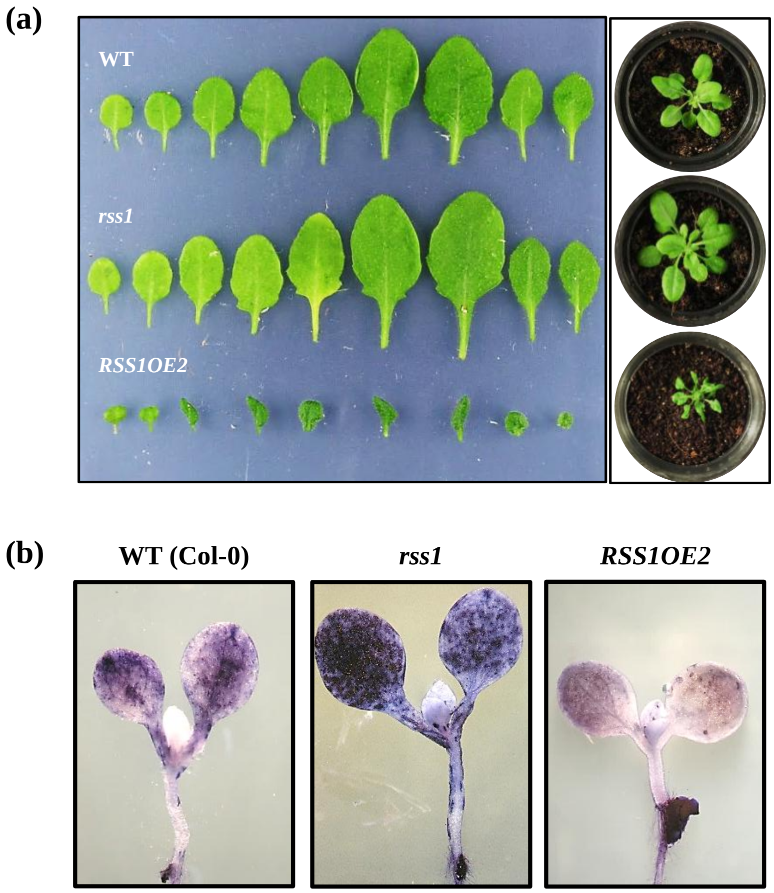

Figure S11. Effect of RSS1 on auxin accumulation.
(a) Auxin overproduction phenotypes of *rss1* plants. **(b)** *in situ* IAA accumulation analysis in WT, *rss1* mutant and *RSS1OE lines*. The blue-purple color in the images displays free IAA immunolocalization signal.

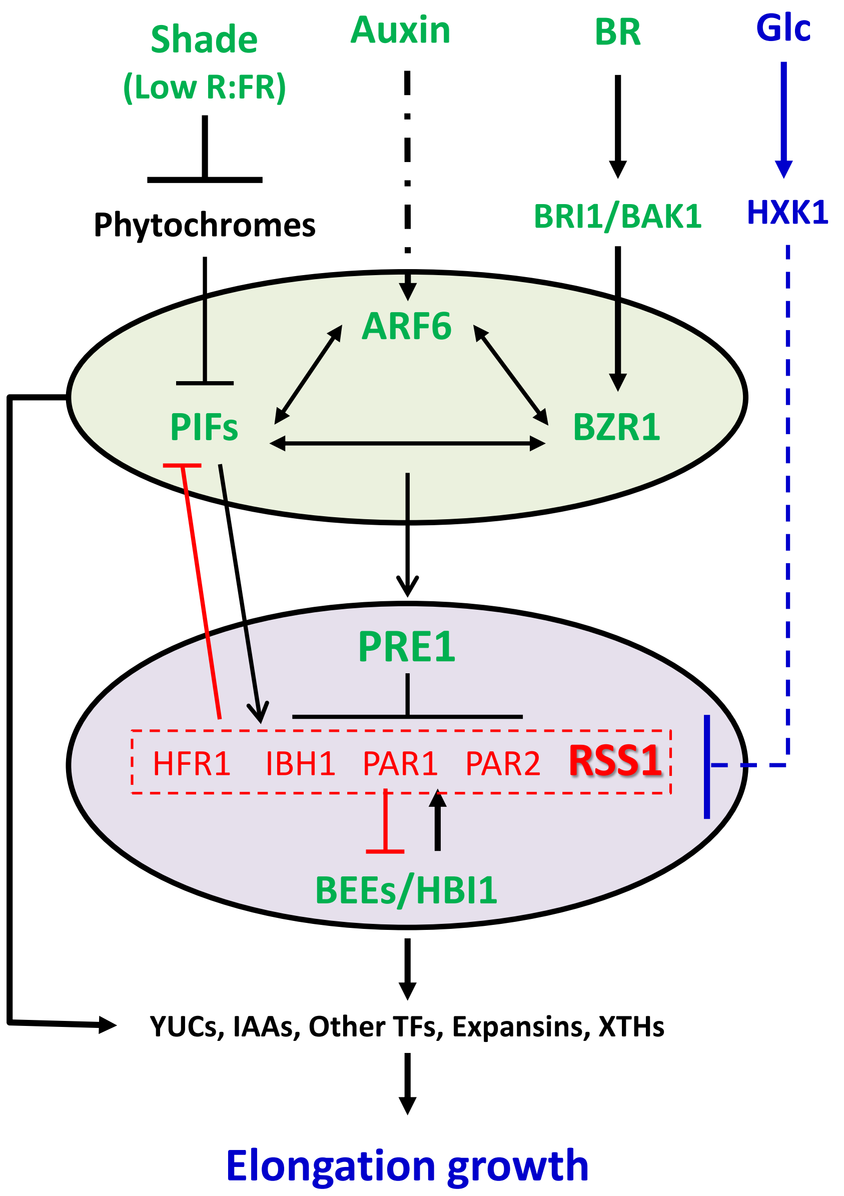

Figure S12. A testable model of RSS1 action and Glc mediated regulation of elongation growth in response to various endogenous and environmental signals in *Arabidopsis*.

In the central growth regulatory B-A-P module, BR-regulated transcription factor BZR1, auxin-regulated transcription factor ARF6, and light/shade/temperature-regulated transcription factor PIF4 interact with each other and control the transcription of common target genes required for cellular elongation growth. Both exogenously applied as well as photosynthetically generated Glc signals might work parallel to- and interact with- auxin, BR and light signals. A tri-antagonistic HLH/bHLH module works further downstream of the B-A-P module and consists of non-DNA binder HLH factors such as PRE1, IBH1/ PAR1 and DNA-binder bHLH factors, such as HBI. The balance between B-A-P and HLH–bHLH factor activities through both positive as well as negative feedback interactions coordinates cell elongation transcriptome downstream to multiple environmental signals. The HLH factor RSS1 identified in present study also form a part of tri-antagonistic loop downstream of B-A-P module and might regulate PIF/HBI1 activity through negative feedback regulation. Green represents positive factors whereas red represents negative factors for elongation growth; dotted lines represent the possibility of additional routes and other factors.

S.no.	Gene name	Forward (5ʹ ---- 3ʹ)	Reverse (5ʹ ---- 3ʹ)
1.	***SALK_043980.52.90.x (LP, RP)***	AAACCATATCGGTTAATCCGG	GATATACCCCCAAATTGGTGG
2.	***SALKseq_043980.2 (LP, RP)***	CACACAAACCTCCCATATTGG	GCCTTTTGCAGGAGAAATTTC
3.	***SALKseq_043980.201 (LP, RP)***	TGATGGCTTATCTTGATTCCG	CCCCTTGATCAATCAATGATG
4.	***SALKseq_043980.0 (LP, RP)***	TCCTCGCAAGTCTAACTCTGC	TGAACTTTGGTGGTTGTTTCG
5.	***LBb1.3***	ATTTTGCCGATTTCGGAAC	
6.	***RSS1 FL directional for YFP fusion***	CACCATGAGAACCTTAAAGACTCA	TAAAACAACATCTTTCTTCTGATC
7.	***RSS1 Promoter***	ATGCCTATATTAACCTGAAATATCC	CTTTTTCAAGAAAATGAGAGAGAGA
8.	***RSS1 FL Y2H***	CATATGATGAGAACCTTAAAGACTCA	CCCGGGTTATAAAACAACATCTTTCT
9.	***RSS1 35s CDS***	ATGAGAACCTTAAAGACTCAGAC	TTATAAAACAACATCTTTC
10.	***RSS1 (qRT-PCR)***	GAGAACCTTAAAGACTCAGACCACAA	CAGCACGTGTGTAAAACTCTCGTA
11.	***18s***	TGCAACAAACCCCGACTTATG	CCCGCGTCGACCTTTTATC
12.	***BEE2***	CAGAGGAAACCAATCCAAAGATCT	CTACAATGTACAAAAAAAGGAATTGCT ATG
13.	***HBI1***	TGCCTGGATGCAATAAGGTCACA	TGGAGCTTCGATAGATGTCGTTTGG
14.	***YUC1***	GATCGCTGGCGAAATTGGT	TCCTGGTGGACCCCTTGA
15.	***YUC2***	GGAACCTTACATAAATGCATCCACTA	CGATTTGGCAAGTTTGGATTG
16.	***YUC3***	CATAAAAAAGCCACATCTTCCTAAAA	AGCCCTCTCCTCGGAAGAAA
17.	***YUC5***	CACCGGCAGATATATTCCATCTC	TGGCGCATCAAGACAACATC
18.	***YUC6***	AAAGTGGGTTATAAGCATGCAAAGA	TGATCATCTGCCCCAAAGG
19.	***YUC9***	GGCATGGAAGTCTCTCTTGATCTT	CGGTAAAACATGAACCGAGCTT
20.	***IAA19***	CGGATGCTACCGGGTTTG	TGCTTCTTGTTCAAGTCATCATCA
21.	***IAA29***	CCGTGTGCATATACAAGATGTTTG	AGAGGAAAAAGATCGAGTGGAAATT
22.	***HFR1***	TGCGAAGGAGGATTTATTGG	GAAACCTTGTCCGTCTTGTG
23.	***ATHB2***	AAATCCATCTGTTTCTGTTACTCCTT	TGTGACGAATCTGAGTTTGGA
24.	***EXP8***	GCCGTGTTCGTCCCGTAA	CATGGGCGGAGCTTGTG
25.	***XTH15***	CGGCACCGTCACTGCTTAC	GAAACTCAAAGTCTATCTCGTCATGTG

Figure S13. List of primers used in this study.
